# A revised version of the Cathcart & El-Jahel model and its application to CDS market

**DOI:** 10.1007/s10203-021-00350-x

**Published:** 2021-08-07

**Authors:** Davide Radi, Vu Phuong Hoang, Gabriele Torri, Hana Dvořáčková

**Affiliations:** 1 Department of Economics and Management, University of Pisa, Pisa, Italy; 2 Department of Finance, VS̆B–Technical University of Ostrava, Ostrava, Czech Republic; 3 Department of Management, Economics and Industrial Engineering, Politecnico di Milano, Milano, Italy; 4 Department of Economics, University of Bergamo, Bergamo, Italy

**Keywords:** Credit risk, Hybrid models, Credit default swaps

## Abstract

The paper considers the pricing of credit default swaps (CDSs) using a revised version of the credit risk model proposed in Cathcart and El-Jahel (2003). Default occurs either the first time a signaling process breaches a threshold barrier or unexpectedly at the first jump of a Cox process. The intensity of default depends on the risk-free interest rate, which follows a Vasicek process, instead of a Cox-Ingersoll-Ross process as in the original model. This offers two advantages. On the one hand, it allows us to account for negative interest rates which are recently observed, on the other hand, it simplifies the formula for pricing CDSs. The goodness of fit of the model is tested using a dataset of CDS credit spreads related to European companies. The results obtained show a rather satisfactory agreement between theoretical predictions and market data, which is identical to the one obtained with the original model. In addition, the values of the calibrated parameters result to be stable over time and the semi-closed form solution ensures a very fast implementation.

## Introduction

Default is the main source of risk in the financial markets. To reduce the exposition to the risk of default investors use credit derivatives, such as credit default swaps (CDSs). They are bilateral agreements to transfer credit risk (on a reference entity) between two parties. The pricing of these derivatives requires to assess credit risk. For this purpose, the financial literature offers three types of models: structural models, reduced-form models and hybrid models.

Structural models evaluate the risk of default of a firm issuing a debt based on one or more variables related to its capital structure, see, e.g., Merton (1974), Black and Cox (1976), Longstaff and Schwartz (1995) and Feng and Volkmer (2012). According to these models, a firm goes bankrupt only if it is in financial distress. Excluding sophisticated versions that accommodate unexpected jumps in the value of firm’s assets and for which analytical tractability is lost, see, e.g., Zhou (2001), structural models are built on the hypotheses that firm’s assets follow a diffusion process and investors are able to observe the true distance to default. These two assumptions make bankruptcy predictable, see Giesecke (2006). In reality, however, the hypothesis of complete information is often violated and investors ask for a premium to compensate the risk of a non-predictable default. As a consequence of this, structural models underestimate short-term credit spreads as confirmed by empirical studies, see, e.g., Jones et al. (1984) and Franks and Torous (1989).

The mechanism that triggers default is different in a reduced-form model. Fully embracing the hypothesis of incomplete information and neglecting any knowledge about the capital structure of a firm, bankruptcy occurs as the first jump of a counting process, see, e.g., Jarrow and Turnbull (1995), Lando (1998), Duffee (1999), Duffie and Singleton (1999), Madan and Schoutens (2008), Schoutens and Cariboni (2009), and Fontana and Montes (2014). The possibility to replicate high credit spreads even for short-term maturities and the mathematical tractability are the main advantages of this approach. Considering default as an exogenous event, dependent only on latent variables, is the main drawback.

Structural and reduced-form approaches can be combined together. The resulting hybrid models offer several advantages, such as mathematical tractability, ability to reproduce high short-term spreads and a structural definition of default. A first example of hybrid model is proposed in Madan and Unal (1998). Employed in Ballestra et al. (2017) for pricing CDSs, it assumes that default occurs at the first jump of a Poisson process as in reduced-form models. However, the intensity of default accounts for structural informations. A similar model is proposed in Madan and Unal (2000), where the intensity of default is approximated by a linear function of the firm’s equity value and of a stochastic risk-free interest rate. Similar is also the structural hazard-rate model in Das and Sundaram (2007), where the equity value follows a constant elasticity of variance process instead of a simpler geometric Brownian motion.^1^ Starting from the different angle offered by the first-passage default models, Duffie and Lando (2001) and Giesecke (2006) obtain a family of hybrid models by introducing incomplete information about firm’s value and default barrier. A more sophisticated hybrid model is proposed in Cathcart and El-Jahel (2006), where default occurs either when a signaling variable breaches a lower barrier as in structural models or at the first jump of a counting process as in Madan and Unal (2000). The intensity of default of the counting process is a linear function of the risk-free interest rate and of the signaling variable measuring credit quality. The short-term interest rate follows a Cox-Ingersoll-Ross (CIR) process, see Cox et al. (1985), while the signaling variable follows a geometric Brownian motion.

In Ballestra et al. (2020), the hybrid model in Cathcart and El-Jahel (2006) has been empirically tested by employing a dataset of CDS spreads. The full-fledged version of this model does not allow to have a closed-form solution for pricing credit risk. To simplify the CDS pricing formula, the hazard rate can be assumed independent of the signaling variable as in Cathcart and El-Jahel (2003), and to simplify it even more, the hazard rate can be assumed constant. In the latter case, the pricing formula for CDS spreads is available analytically except for the numerical approximation of a univariate integral. The empirical analysis in Ballestra et al. (2020) underlines that the model with constant hazard rate provides a good compromise between goodness of fit and computational efficiency.

In this work, we extend the analysis in Ballestra et al. (2020) by proposing a revised version of the model in Cathcart and El-Jahel (2003) where the short-term interest rate follows a Vasicek process instead of a CIR process.^2^ In assessing companies’ credit risk, this allows us to account for the impact of negative interest rates frequently observed in the last decade. Then, we derive a closed-form solution for the default probability and a formula for pricing CDS spreads in semi-closed form. A one-dimensional integral only needs to be approximated numerically.

As confirmed by a numerical test, the computational burden of the CDS pricing formula obtained is equal to the one of the CDS pricing formula obtained in Ballestra et al. (2020) by imposing a constant hazard rate. Respect to this latter model, however, the current CDS pricing formula must ensure a superior empirical fitting, which we expect to be similar to the one obtained with the model proposed in Cathcart and El-Jahel (2003). To test this conjecture, a calibration procedure using econometric techniques is employed. This procedure is based on the maximum-likelihood estimation of the parameters of the process used to model the short-term default-free interest rate. Then, by minimizing the mean absolute percentage error, we observe that the fitting of the empirical term structure of the CDS spreads obtained with our model is identical to the one obtained with the credit risk model in Cathcart and El-Jahel (2003). This result corroborates our conjecture and allows us to conclude that the credit risk model considered here is a valid alternative to the model proposed in Cathcart and El-Jahel (2003). Indeed, it ensures the same goodness of fit but it takes a hundred times less CPU time to compute a CDS spread.

In addition, a dataset of CDS spreads observed in the last three years is employed to show the stability over time of the calibrated values of the parameters of the new credit risk model considered here. This analysis is performed by considering the spreads of CDSs written on 142 companies. The companies are divided in three groups according to their S&P rating. The model reveals that companies with high credit rating have a higher equity value, their credit quality signaling variable is less volatile, their hazard rate is lower and their duration gap is positive while it becomes negative when companies with lower credit rating are considered. We also study how the calibration varies across different sectors by grouping the companies according to the two digits NAICS sector code, finding that there aren’t relevant differences in the calibrated parameters across sectors. Finally, we observe that the rise of CDS spreads in conjunction with the COVID-19 pandemic is mainly capture by the intensity-of-default parameters.

The road map of the paper is the following. Section 2 introduces the valuation framework proposed. Section 3 derives formulas in closed form for computing the probability of default, the current price of a survival security and the forward price of a survival security. These results are employed in Sect. 4, where two formulas for pricing CDS spreads are derived. The first one obtained assuming the current valuation framework. The second one obtained assuming the credit risk model in Cathcart and El-Jahel (2003). The main differences between the two pricing formulas are discussed. In Sect. 5 an empirical analysis estimates the goodness of fit of the two credit risk models in replicating the curve of CDS spreads. In Sect. 6 an investigation employing historical data shows the stability over time of the calibrated values of the parameters of the proposed credit risk model. Section 7 concludes. Appendix A recaps the credit risk model in Cathcart and El-Jahel (2003). All proofs are in Appendix B. Appendix C contains the list of the companies studied, the descriptive statistics of the CDS curves, and the results of the calibrations for selected companies.

## The valuation framework

A credit-risk modeling framework is developed by assuming that the market is frictionless and perfectly competitive. Trading takes place continuously. Investors act as price takers and there are no taxes, transaction costs, or informational asymmetries.

**Fig. 1:**
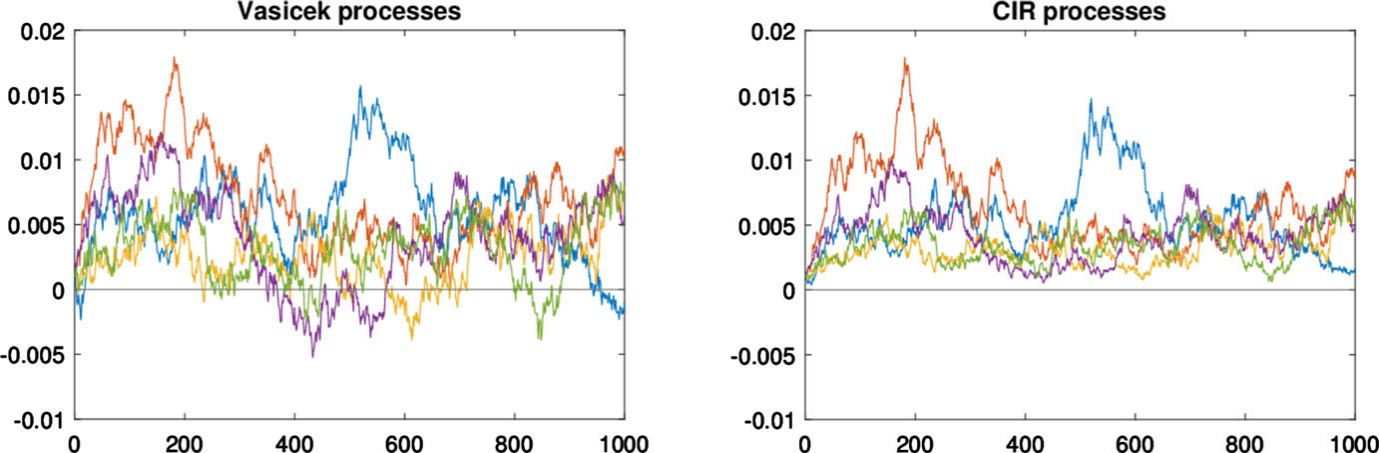
Simulated Vasicek and CIR stochastic processes. Parameters for Vasicek: $k=1$, $\mu =0.005$, $\sigma _r\left( t_{0}\right) =0.005$, $r=0.001$. Parameters for CIR: $k=1$, $\mu =0.005$, $\sigma _r=0.05$, $r=0.001$. The process is simulated using a Euler scheme with discrete time steps $\Delta t = 0.01$. For the two models we used the same Wiener process as driver to make the visual comparison between the two models easier

The default-free interest rate is supposed to evolve according to the following process

1 $$\begin{aligned} \text {d}r\left( t\right) =k\left( \mu -r\left( t\right) \right) \text {d}t+\sigma _{r}\left( t,r\left( t\right) \right) \text {d}W_{r}\left( t\right) \end{aligned}$$

where *k* is a real parameter that measures the speed at which the default-free interest rate converges to the long-run average $\mu$ and $W_{r}$ is a standard Wiener process under the martingale measure *Q*. Concerning the diffusion term, in this work we assume that $\sigma _{r}\left( t,r\left( t\right) \right) =\sigma _{r}$. That is, we assume that the default-free interest rate follows a Vasicek process (Vasicek 1977). In contrast, Cathcart and El-Jahel (2003), Cathcart and El-Jahel (2006), and Ballestra et al. (2020) model the short-term interest rate as a Cox-Ingersoll-Ross (CIR) process (i.e. $\sigma _{r}\left( t,r\left( t\right) \right) =\sigma _{r}\sqrt{r\left( t\right) }$, Cox et al. 1985). Considering the short-term interest rate following a Vasicek process represents the main novelty from the modeling point of view of this work. This novelty allows us to account for negative interest rates, which are recently experienced in the European markets and, as discussed later, it gives us significant computational advantages for the calibration. Figure 1 presents a simulation of the Vasicek and CIR stochastic processes obtained using an Euler scheme. The plot highlights how the main difference between the two is the behaviour when the process approaches zero: while the former can assume negative values, the latter remains always positive and reduces its volatility.

Assuming that the short-term riskless rate follows process (1) with $\sigma _{r}\left( t,r\left( t\right) \right) =\sigma _{r}$, the risk-neutral price at the current time $t_{0}$ of a default-free pure discount bond with maturity *T* is given by (see Vasicek 1977):

2 $$\begin{aligned} P_{_{V}}\left( r\left( t_{0}\right) ,t_{0};T\right) =e^{\displaystyle A\left( t_{0};T\right) +B\left( t_{0};T\right) r\left( t_{0}\right) } \end{aligned}$$

where

3 $$\begin{aligned} B\left( t_{0};T\right)= & {} \frac{e^{-k\left( T-t_{0}\right) }-1}{k}\nonumber \\ A\left( t_{0};T\right)= & {} \left( \frac{\sigma _{r}^{2}}{2 k^2 }-\mu \right) \left( B\left( t_{0};T\right) +T-t_{0}\right) -\frac{\sigma _{r}^{2}}{4\kappa }B^{2}\left( t_{0};T\right) \end{aligned}$$

A firm’s credit quality is measured by a signaling variable denoted by *x*.^3^ A higher (lower) value of this signaling variable means a higher (lower) credit quality. Specifically, its risk-adjusted dynamics follows the diffusion process

4 $$\begin{aligned} \text {d}x\left( t\right) =\alpha x\left( t\right) \text {d}t+\sigma _{x} x\left( t\right) \text {d}W_{x}\left( t\right) \end{aligned}$$

where $\alpha$ is a real parameter, $\sigma _{x}$ is a real and positive parameter, while $W_{x}$ is a standard Wiener process under the martingale measure *Q* and is uncorrelated to $W_{r}$.

Given the dynamics of the default-free interest rate and of the signaling variable, default occurs either *expectedly* at the first time the signaling variable *x* breaches from above the threshold value $x_{L}$, also known as the default barrier, or *unexpectedly* at the jump event of a Cox, or counting, process with hazard rate $\lambda \left( r\right) =a+br$. Here, *a* and *b* are real value parameters and $\lambda \left( r\right)$ is imposed to be non-negative. The intensity of default is therefore assumed to be a linear function of the short-term risk-free interest rate. As shown in Madan and Unal (2000), the parameter *b* proxies the duration gap of a company. A positive value of *b* indicates a positive duration gap. In this case, an increase of the interest rate reduces the value of the assets more than the values of the liabilities and the credit spreads are positively related to the short-term interest rate. A negative value of *b* indicates a negative duration gap. In this case, an increase of the interest rate reduces the value of the assets less than the values of the liabilities and the credit spreads are negatively related to the short-term interest rate.

At the default time, the company defaults on all of its obligations and some restructuring occurs so that the assets of the company are allocated exogenously among various bondholders. Specifically, we assume the so-called *recovery of treasury*, see, e.g., Duffie and Singleton (1999) and Guo et al. (2008), according to which bondholders receive $\delta$ default-free discounted bonds with the same maturity and promised payment as for the original security. The recovery rate $\delta$ is assumed constant.

The model that we propose here is closely related to Cathcart and El-Jahel (2003) with the only difference being the dynamics of the default-free interest rate (both models are special cases of Cathcart and El-Jahel 2006). The model of Cathcart and El-Jahel (2003) however is computationally heavy, and Ballestra et al. (2020) suggest to use a constant hazard rate to balance goodness of fit and computational simplicity. Thanks to the different specification of the interest rate process, in our model we can relax the assumption of constant hazard rate, assuming instead that this quantity depends on the short-term interest rate with no additional computational burden.

A possible drawback exists for the current credit-risk modeling framework. Considering the short-term interest rate following a Vasicek process, the linear model for the intensity of default here employed has the potential of yielding negative credit spreads as hazard rates can go negative. However, as also specified in Madan and Unal (2000) this problem can be mitigated, in practice, by calibrating the resulting model to positive credit spread data over a finite horizon of debt maturities.

## The probability to survive

Consider the stopping time $\tau _{x}$, defined as the first time the random variable *x* breaches the default barrier $x_{L}$. Moreover, consider the stopping time $\tau _{r}$, defined as the first time the counting process with hazard rate $\lambda \left( r\right)$ has a jump.

According to the valuation framework in the previous section, $t_{d}=\min \left\{ \tau _{x},\tau _{r}\right\}$ is the random time of default and $Q\left( t_{d}>T\right)$ is the probability to survive up to time *T*. Note that

5 $$\begin{aligned} Q\left( t_{d}>T\right)= & {} \mathbb {E}^{Q}\left[ \mathbf {1}_{\left\{ t_{d}>T\right\} } \right] \nonumber \\= & {} \mathbb {E}^{Q}\left[ \mathbf {1}_{\left\{ \tau _{x}>T\wedge \tau _{r}>T\right\} } \right] \nonumber \\= & {} \mathbb {E}^{Q}\left[ \mathbf {1}_{\left\{ \tau _{x}>T\right\} }\mathbf {1}_{\left\{ \tau _{r}>T\right\} } \right] \nonumber \\&\overset{(I)}{=}&\mathbb {E}^{Q}\left[ \mathbf {1}_{\left\{ \tau _{x}>T\right\} }\right] \mathbb {E}^{Q}\left[ \mathbf {1}_{\left\{ \tau _{r}>T\right\} } \right] \end{aligned}$$

where (I) follows from the fact that *x* and *r* are independent random variables. Then, by Girsanov’s Theorem and reflection principle, see, e.g., (Shreve 2010, pp. 297–299), we have that

6 $$\begin{aligned}&\mathbb {E}^{Q}\left[ \mathbf {1}_{\left\{ \tau _{x}>T\right\} }\right] = f\left( x\left( t_{0}\right) ,t_{0};T\right) \nonumber \\&\quad = N\left( d_{1}\left( T-t_{0}\right) \right) -e^{\left( 1-\frac{2\alpha }{\sigma _{x}^{2}}\right) \ln \frac{x\left( t_{0}\right) }{x_{L}}}N\left( d_{2}\left( T-t_{0}\right) \right) \end{aligned}$$

where

7 $$\begin{aligned} d_{1}\left( s\right) = \frac{\ln \frac{x\left( t_{0}\right) }{x_{L}} +\left( \alpha -\frac{1}{2}\sigma _{x}^{2}\right) s }{\sigma _{x}\sqrt{s}} \end{aligned}$$

and

8 $$\begin{aligned} d_{2}\left( s\right) = \frac{-\ln \frac{x\left( t_{0}\right) }{x_{L}} +\left( \alpha -\frac{1}{2}\sigma _{x}^{2}\right) s}{\sigma _{x}\sqrt{s}} \end{aligned}$$

Employing formula (6), the analytical expression for the probability to survive of a firm and the price of a survival security (defaultable bond with zero recovery) are defined in the following Proposition (proof in Appendix B).

### Proposition 1

Assuming the hybrid credit-risk model in Sect. 2, we have that

9 $$\begin{aligned} Q\left( t_{d}>T\right) = f\left( x\left( t_{0}\right) ,t_{0};T\right) g_{_{V}}\left( r\left( t_{0}\right) ,t_{0};T\right) \end{aligned}$$

where *f* is defined in (6), while

10 $$\begin{aligned} g_{_{V}}\left( r\left( t_{0}\right) ,t_{0};T\right) = e^{\displaystyle E_{1}\left( t_{0};T\right) +E_{2}\left( t_{0};T\right) r\left( t_{0}\right) } \end{aligned}$$

with

11 $$\begin{aligned} E_{1}\left( t_{0};T\right)= & {} \left( \frac{b \sigma _{r}^{2} }{ 2k^2}-\mu \right) b\left( B\left( t_{0};T\right) + \left( T-t_{0}\right) \right) -\frac{b^2 \sigma _{r}^{2}}{4k}B^{2}\left( t_{0};T\right) -a\left( T-t_{0}\right) \nonumber \\ E_{2}\left( t_{0};T\right)= & {} bB\left( t_{0};T\right) \end{aligned}$$

and $B\left( \cdot ;\cdot \right)$ defined as in (3). Moreover, the current price of a survival security with unitary face value and maturity *T* is given by

12 $$\begin{aligned} S_{_{V}}\left( r\left( t_{0}\right) ,x\left( t_{0}\right) ,t_{0};T\right) = f\left( x\left( t_{0}\right) ,t_{0};T\right) \tilde{g}_{_{V}}\left( r\left( t_{0}\right) ,t_{0};T\right) \end{aligned}$$

where $\tilde{g}_{_{V}}$ is obtained by $g_{_{V}}$ substituting *b* with $b+1$.

The price of a survival security in Proposition 1 is the discounted value of the probability to survive when the short-term interest rate follows a Vasicek process.

The formula to price a survival security as well as the probability to survive are both available in closed form as shown in Proposition 1. The forward price of a survival security also admits a closed-form solution. This is a relevant point as the forward price of a survival security is required for pricing CDSs. In the current valuation framework, the forward price of the survival security discounts the promised unit payoff for reasons related to exposure to the hazard of default, as captured by the process for the timing risk of default $\lambda \left( r\right)$, and multiply it for the probability that the signaling variable does not breach the default barrier $x_{L}$.

To compute the forward price of a survival security, we consider the *forward measure* $Q^{T}$ as in Madan and Unal (2000), which is the unique equivalent martingale measure under which the values of traded assets discounted by the price of the default-free bond, in this case $P_{_{V}}\left( r\left( t_{0}\right) ,t_{0};T\right)$, are martingales.

The change of measure density process from *Q* to $Q^{T}$ is given by the Radon-Nikodym derivative which is equal to the price of a default-free bond with maturity *T*, see, e.g., Brigo and Mercurio (2007) and Madan and Unal (2000). Then, applying Girsanov’s theorem, the $Q^{T}$ dynamics for *x* and *r* are given by

13 $$\begin{aligned} \text {d}x\left( t\right)= & {} \alpha x\left( t\right) \text {d}t+\sigma _{x} \text {d}W_{x}\left( t\right) \nonumber \\ \text {d}r\left( t\right)= & {} \left( \mu \left( k-r\left( t\right) \right) - \sigma _{x}^{2} B\left( t;T\right) \right) \text {d}t + \sigma _{r} d\tilde{W}_{r}\left( t\right) \end{aligned}$$

where $W_{x}\left( t\right)$ and $\tilde{W}_{r}\left( t\right)$ are Wiener processes under $Q^{T}$ while $B\left( t;T\right)$ is defined in (3). Since by assumption the signaling variable is uncorrelated to the short-term interest rate, its dynamics does not change when we move from the martingale measure *Q* to the forward measure $Q^{T}$, i.e. $Q^{T}\left( \tau _{x}>T\right) =Q\left( \tau _{x}>T\right)$.

The formula in closed form for the forward price of a survival security is provided in following proposition (proof in Appendix B).

### Proposition 2

Assuming the hybrid credit-risk model in Sect. 2, the forward price of a survival security with unitary face value and maturity *T* is given by

14 $$\begin{aligned} F_{_{V}}\left( x\left( t_{0}\right) ,r\left( t_{0}\right) ,t_{0};T\right)= & {} Q^{T}\left( t_{d}>T\right) \nonumber \\= & {} f\left( x\left( t_{0}\right) ,t_{0};T\right) h_{_{V}}\left( r\left( t_{0}\right) ,t_{0};T\right) \end{aligned}$$

where *f* is defined in (6), while

15 $$\begin{aligned} h_{_{V}}\left( r\left( t_{0}\right) ,t_{0};T\right) = e^{\displaystyle E_{3}\left( t;T\right) +E_{4}\left( t;T\right) r\left( t_{0}\right) } \end{aligned}$$

with

16 $$\begin{aligned} E_{3}\left( t_{0};T\right)= & {} b\left( \frac{\left( 1+\frac{b}{2}\right) \sigma _{r}^{2}}{k^2}-\mu \right) \left( B\left( t_{0};T\right) +\left( T-t_{0}\right) \right) \nonumber \\&+\left( 1+\frac{b}{2}\right) \frac{b\sigma _{r}^{2}}{2k}B^{2} \left( t_{0};T\right) -a\left( T-t_{0}\right) \nonumber \\ E_{4}\left( t_{0};T\right)= & {} bB\left( t_{0};T\right) \end{aligned}$$

and $B\left( \cdot ;\cdot \right)$ defined as in (3).

## Pricing credit default swaps

In this section, we consider the problem of pricing CDSs. To this aim, let us consider a CDS written on a unit bond with initial protection time $t_{0}$, final protection time *T* ($T-t_{0}$) is therefore the time to maturity of the CDS), and random time of default $t_{d}$. Moreover, let us denote a CDS spread by $\Pi$ and let us assume that it is paid continuously. Then, at time $t_{0}$ the premium leg is given by (see e.g., Brigo and Mercurio 2007, p. 736 and Gündüz and Uhrig-Homburg 2014)

17 $$\begin{aligned} \text {PremiumLeg}=\mathbb {E}^{Q}\left[ \Pi \int _{t_{0}}^{T}e^{ -\int _{t_{0}}^{z}r\left( u\right) du}\mathbf {1}_{\left\{ t_{d} >z\right\} }dz\right] \end{aligned}$$

where $\mathbf {1}_{\left\{ \cdot \right\} }$ denotes the indicator function. Moreover, let *LGD* denote the single protection payment (so-called loss given default), operated at the time of default $t_{d}$. According to the assumption of recovery of treasury with recovery rate $\delta$, see again Guo et al. (2008), we have:

18 $$\begin{aligned} LGD = \left( 1-\delta \right) P\left( r\left( t_{d}\right) ,t_{d};T\right) \end{aligned}$$

where $P\left( r\left( t_{d}\right) ,t_{d};T\right)$ is the price at time $t_{d}$ of a default-free discount bond with maturity *T*. Therefore, the protection leg is given by

19 $$\begin{aligned} \text {ProtectionLeg}= P\left( r\left( t_{0}\right) ,t_{0};T\right) \mathbb {E}^{Q^{T}}\left[ \left( 1-\delta \right) \mathbf {1}_{\left\{ T \ge t_{d} >t_{0} \right\} } \right] \end{aligned}$$

where $Q^{T}$ is the forward measure discussed above. By equating the premium leg to the protection leg and solving for $\Pi$, we obtain the so-called CDS par spread (hereafter simply referred to as CDS spread) at the initial protection time $t_{0}$:

20 $$\begin{aligned} \Pi \left( x\left( t_{0}\right) ,r\left( t_{0}\right) ,t_{0};T\right) = \frac{P\left( r\left( t_{0}\right) ,t_{0};T\right) \mathbb {E}^{Q^{T}}\left[ \left( 1-\delta \right) \mathbf {1}_{\left\{ T \ge t_{d}>t_{0} \right\} } \right] }{\mathbb {E}^{Q}\left[ \displaystyle \int _{t_{0}}^{T}e^{ -\int _{t_{0}}^{z}r\left( u\right) du}\mathbf {1}_{\left\{ t_{d} >z\right\} }dz\right] } \end{aligned}$$

Assuming the credit risk model in Sect. 2, a solution in semi-closed form for computing the CDS spread in (20) is proposed in the following Proposition (see the proof in Appendix B)

### Proposition 3

Assuming the credit risk model in Sect. 2, the CDS spread in (20), that we denote by $\Pi _{_{V}}$, can be computed as follows:

21 $$\begin{aligned} \Pi _{_{V}}\left( x\left( t_{0}\right) ,r\left( t_{0}\right) ,t_{0};T\right) = \frac{\displaystyle P_{_{V}}\left( r(t_{0}),t_{0};T\right) \left( 1-\delta \right) \left( 1-F_{_{V}}\left( x\left( t_{0}\right) ,r\left( t_{0}\right) ,t_{0};T\right) \right) }{\displaystyle \int _{t_{0}}^{T}S_{_{V}}\left( x\left( t_{0}\right) ,r\left( t_{0}\right) ,t_{0};s\right) ds} \end{aligned}$$

where $F_{_{V}}\left( \cdot \right)$ is the forward price of a survival security defined in Proposition 2, $S_{_{V}}\left( \cdot \right)$ is the current price of a survival security defined in Proposition 1, while $P_{_{V}}\left( \cdot \right)$ is the price of a default-free pure discount bond defined as in (2).

The one-dimensional integral at the denominator of the pricing formula (21) needs to be approximated numerically. The numerical approximation is obtained by employing a 32-points Newton-Cotes quadrature rule. A numerical test indicates that this one-dimensional differential quadrature approximation ensures the required level of precision when pricing CDSs with maturities lower than or equal to 30 years.^4^

To test the validity of the valuation framework proposed, the pricing of CDS spreads is also addressed with the credit risk model of Cathcart and El-Jahel (2003). The pricing formula for CDS spreads under the Cathcart and El-Jahel (2003) model is provided in Ballestra et al. (2020) and is recap in the following Proposition:^5^

### Proposition 4

Assuming the credit risk model in Cathcart and El-Jahel (2003), the CDS spread in (20), that we denote by $\Pi _{_{CIR}}$, can be computed as follows:

22 $$\begin{aligned}&\Pi _{_{CIR}}\left( x\left( t_{0}\right) ,r\left( t_{0}\right) ,t_{0};T\right) \nonumber \\&\qquad = \frac{\displaystyle P_{_{CIR}}\left( r(t_{0}),t_{0};T\right) \left( 1-\delta \right) \left( 1-F_{_{CIR}}\left( x\left( t_{0}\right) ,r\left( t_{0}\right) ,t_{0};T\right) \right) }{\displaystyle \int _{t_{0}}^{T} S_{_{CIR}}\left( x\left( t_{0}\right) ,r\left( t_{0}\right) ,t_{0};s\right) ds} \end{aligned}$$

where $F_{_{CIR}}\left( \cdot \right)$ is the forward price of a survival security defined in Proposition 6, $S_{_{CIR}}\left( \cdot \right)$ is the current price of a survival security defined in Proposition 5, while $P_{_{CIR}}\left( \cdot \right)$ is the price of a default-free pure discount bond defined as in (24).

To compute the pricing formula (22) the one-dimensional integral at the denominator needs to be approximated numerically. As for the pricing formula (21), the 32-points Newton-Cotes quadrature rule can be employed. In addition to this, the forward price of a survival security must also be approximated numerically when computing (22), see Proposition 6 in Appendix A. Specifically, a numerical approximation of the solution of the system of two ordinary differential equations in (33) is required when pricing a CDS spread under the credit risk model in Cathcart and El-Jahel (2003). Such approximation is obtained employing the fourth-order-Runge-Kutta method.

**Table 1:** CPU time for obtaining a single CDS spread

	$\Pi _{_{V}}$	$\Pi _{_{CIR}}$
CPU time	$2.407\times 10^{-4} \ s$	$0.022\ s$

The credit risk model here proposed offers therefore an advantage in terms of computational complexity. This advantage is due to the forward price of a survival security, available in closed form for the valuation framework here proposed and not available in closed form for the credit risk model in Cathcart and El-Jahel (2003).

The different computational complexity impacts on the CPU time required to price a CDS spread. Table 1 indicates that a single CDS spread is computed in around two hundredths of a second when the credit risk model in Cathcart and El-Jahel (2003) is employed. The same single CDS spread requires about a hundredth less CPU time when the valuation framework here proposed is employed. This difference is estimated by using a computer Intel Core i7 CPU, 2.3 GHz, with all the software codes written in Matlab 9.3, and becomes particularly relevant in the calibration phase. In fact, generally tens of thousands of CDS spreads need to be computed for a single calibration.

**Fig. 2:**
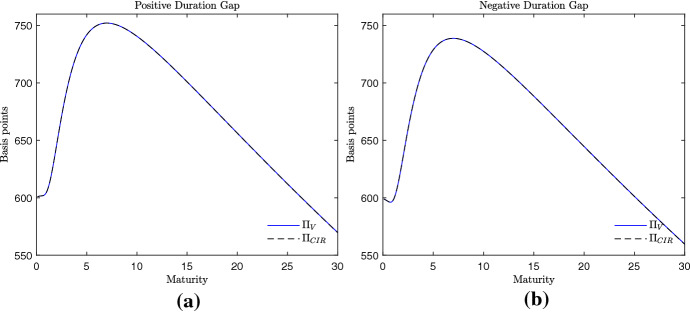
Term structure of the CDS spreads. Example 1, parametrization with humped curve. Parameters: $\frac{x}{x_{L}}=2$, $\alpha = 0.01$, $\sigma _{x}=0.2$, $r=0.001$, $k=1$, $\mu =0.015$, $\sigma _{r}=0.005$, $a=0.1$, $\delta = 0.4$. Positive duration gap: $b=0.1$ (**a**). Negative duration gap: $b=-0.1$ (**b**)

**Fig. 3:**
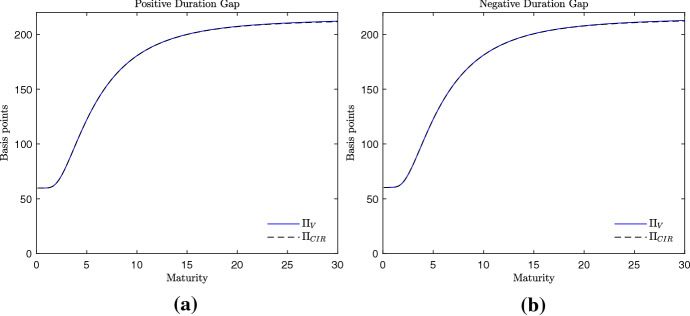
Term structure of the CDS spreads. Example 2, parametrization with upward sloping curve. Parameters: $\frac{x}{x_{L}}=2.5$, $\alpha = 0.01$, $\sigma _{x}=0.2$, $r=-0.005$, $k=0.170$, $\mu =0.005$, $\sigma _{r}=0.003$, $a=0.01$, $\delta = 0.4$. Positive duration gap: $b=0.01$ (**a**). Negative duration gap: $b=-0.01$ (**b**). The interest rate parameters are as in Table 2

**Fig. 4:**
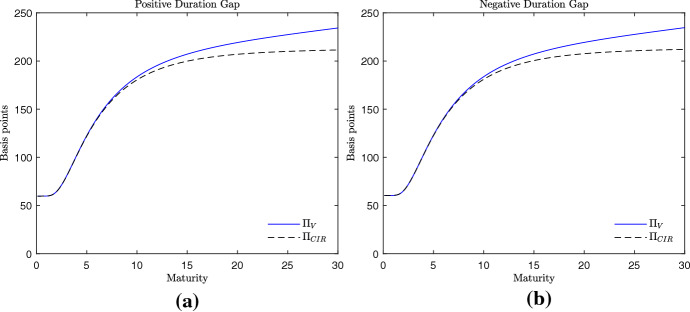
Term structure of the CDS spreads. Example 3, parametrization with upward sloping curve and high interest rate volatility $\sigma _{r}$. Parameters: $\frac{x}{x_{L}}=2.5$, $\alpha = 0.01$, $\sigma _{x}=0.2$, $r=-0.005$, $k=0.170$, $\mu =0.005$, $\sigma _{r}=0.02$, $a=0.01$, $\delta = 0.4$. Positive duration gap: $b=0.01$ (panel (**a**)). Negative duration gap: $b=-0.01$ (panel (**b**))

Despite the different computational complexity, the term structure of CDS spreads obtained with the pricing formulas (21) and (22) are very similar. In Fig. 2, for example, two humped curves of CDS spreads are proposed, one obtained assuming a positive duration gap (panel (a)) and one obtained assuming a negative duration gap (panel (b)). For maturities from zero to 30 years, the CDS spreads generated by the credit risk model in Cathcart and El-Jahel (2003) are equal to the ones generated by the valuation framework here proposed. The same occurs when upward sloping curves of CDS spreads are considered. It is the case of Fig. 3, where the employed values of the interest rate parameters are the ones used in the empirical analysis in the next section. Even in this case we observe that the two credit risk models generate similar CDS spreads independently of the sign of the duration gap. This suggests that the two models predict almost equal credit spreads. The only differences can be observed when the volatility parameter of the short-term interest rate $\sigma _{r}$ takes large values (Fig. 4). In this case, we observe that the CDS spreads related to long-term maturities are higher when generated by the credit risk model here proposed. However, such a difference is observed for values of $\sigma _{r}$ which are far from the ones observed in reality. Therefore, we can conclude that the two models generate almost equal curves of CDS spreads and the goodness of fit of the two models should be similar. This conjecture is corroborated by the empirical analysis that follows.

## Empirical testing of models

We use a dataset of (single-name) CDSs. We consider the credit spreads of CDSs written on bonds issued by 142 European companies. For each company, ten different maturities of CDSs are considered, that is $T_{1}=6$ months, $T_{2}=1$ year, $T_{3}=2$ years, $T_{4}=3$ years, $T_{5}=4$ years, $T_{6}=5$ years, $T_{7}=7$ years, $T_{8}=10$ years, $T_{9}=20$ years, and $T_{10}=30$ years. The CDS data are obtained from the Thomson Reuters Eikon database. Following Packer and Zhu (2005) and consistently with the assumption of recovery of treasury, we only include CDSs with restructuring clause XR14, i.e. *no restructuring*. Furthermore, we only collect CDSs written on debts classified as *senior unsecured* and traded in Euros. The list of all the 142 companies considered in the analysis and the descriptive statistics of the CDS spread curves are available in Appendix C.

Moreover, we also use a dataset of monthly quotes of the Euribor rate. In particular, we consider the monthly observations of the 1-week Euribor rate that we use as a proxy for the default-free interest rate.

These datasets are employed to conduct an empirical investigation. The goal of this investigation is to compare the goodness of fit of the pricing formulas in Propositions 3 and 4, obtained assuming the current credit risk model and the one in Cathcart and El-Jahel (2003), respectively.

To this aim, we estimate the parameters of the CIR model (adopted in Cathcart and El-Jahel (2003) to describe the dynamics of the risk-free interest rate) and of the Vasicek model (adopted in the valuation framework in Sect. 2 to describe the dynamics of the risk-free interest rate) by employing the maximum-likelihood method, see, e.g., Fergusson and Platen (2015) and Ballestra et al. (2016), and using the monthly observations of the 1-week Euribor rate from February 1, 2010 to February 3, 2020. Moreover, $r\left( t_{0}\right)$ is set equal to the 1-week Euribor rate at the day at which the CDS spreads are considered, that is February 3, 2020. The current time $t_{0}$ is set equal to 0 for notational simplicity and the estimated values of *k*, $\mu$ and $\sigma _{r}^{2}$ for the two models employed are reported in Table 2.

**Table 2:** Maximum-likelihood estimation of the parameters of the CIR model, first line, and of the parameters of the Vasicek model, second line

	*k*	$\mu$	$\sigma _{r}$
CIR	0.0170	$-\,0.0049$	0.0031
Vasicek	0.0170	$-\,0.0049$	0.0029

Monthly observations of the 1-week Euribor rates for the period from February, 2010, to February, 2020, are considered

To complete the calibration of the credit risk models, the values of the interest rate parameters in Table 2 are employed and the recovery rate $\delta$ is set to 0.4. A constant recovery rate equal to $40\%$ is a common assumption in the literature, see e.g. Altman and Kishore (1996), Jankowitsch et al. (2008) and Madan (2014). Then, for each company considered, the remaining parameters of the credit risk models are obtained by fitting the term structure of realized CDS spreads quoted on February 3, 2020. This is done by using a calibration procedure analogous to the one proposed in Hao et al. (2013), Ballestra et al. (2017) and Ballestra et al. (2020). Specifically, the parameters of the model that still need to be estimated, namely $\frac{x_{0}}{x_{L}}$, $\alpha$, $\sigma _{x}^{2}$, *a* and *b*, are obtained by minimizing the mean absolute percentage error (MAPE) between the empirical CDS spreads and the theoretical ones:

23 $$\begin{aligned} \text {MAPE}\left( \frac{x_{0}}{x_{L}},\alpha ,\sigma _{x}^{2},a,b\right) =\frac{1}{10}\sum \limits _{i=1}^{10}\left| \frac{\Pi ^{*}\left( 0,T_{i}\right) - \Pi _{j} \left( x_{0},r\left( 0\right) ,0;T_{i}\right) }{\Pi ^{*}\left( 0,T_{i}\right) }\right| \end{aligned}$$

where $\Pi _{j}\left( x_{0},r\left( 0\right) ,0;T_{i}\right)$ is evaluated using the CDS pricing formula (21) if $j=V$ and the CDS pricing formula (22) if $j=CIR$, while $\Pi ^{*}\left( 0,T_{i}\right)$ denotes the value observed at time 0 (February 3, 2020) of the spread of a CDS with time to maturity $T_{i}$.

**Table 3:** Statistics of the calibrated values and of the mean absolute percentage error (goodness of fit) for the credit risk model in Cathcart and El-Jahel (2003), formula $\Pi _{_{CIR}}$, and for its revised version here proposed, formula $\Pi _{_{V}}$

			$\frac{x_{0}}{x_{L}}$	$\alpha$	$\sigma _{x}$	*a*	*b*	MAPE %
(AAA) – (A-)	$\Pi _{CIR}$	Average	3.224	0.054	0.237	0.010	1.516	$8.53\%$
		Min	2.392	0.023	0.177	$-0.013$	$-2.752$	$2.39\%$
		Max	4.715	0.120	0.300	0.010	1.873	$19.20\%$
		SD	0.589	0.020	0.032	0.003	0.667	$2.48\%$
	$\Pi _{V}$	Average	3.224	0.054	0.237	0.010	1.506	$8.52\%$
		Min	2.392	0.022	0.177	$-0.013$	$-2.804$	$2.25\%$
		Max	4.716	0.121	0.300	0.010	1.860	$19.23\%$
		SD	0.588	0.020	0.032	0.003	0.672	$2.51\%$
(BBB+) – (BBB-)	$\Pi _{CIR}$	Average	3.002	0.037	0.236	0.009	1.285	$8.10\%$
		Min	1.612	0.009	0.101	$-0.009$	$-3.832$	$0.90\%$
		Max	5.076	0.191	0.300	0.010	1.832	$15.73\%$
		SD	0.569	0.024	0.030	0.003	1.069	$2.81\%$
	$\Pi _{V}$	Average	2.999	0.037	0.236	0.009	1.277	$8.06\%$
		Min	1.612	0.009	0.101	$-0.009$	$-3.881$	$0.31\%$
		Max	4.977	0.188	0.300	0.010	1.822	$15.76\%$
		SD	0.558	0.023	0.030	0.003	1.075	$2.92\%$
(BB+) – (CCC)	$\Pi _{CIR}$	Average	2.589	0.085	0.243	0.005	$-3.392$	$6.79\%$
		Min	1.015	$-0.029$	0.115	$-0.020$	$-19.872$	$3.42\%$
		Max	5.035	1.314	0.294	0.010	1.639	$10.41\%$
		SD	0.849	0.317	0.047	0.010	7.282	$2.18\%$
	$\Pi _{V}$	Average	2.583	0.084	0.244	0.005	$-3.434$	$5.70\%$
		Min	1.015	$-0.030$	0.114	$-0.020$	$-19.946$	$1.86\%$
		Max	4.936	1.304	0.300	0.010	1.638	$10.19\%$
		SD	0.831	0.315	0.047	0.010	7.351	$2.39\%$

For each one of the 142 European companies listed in Appendix C, the parameters are calibrated by employing CDS spreads with maturities 6 months and 1, 2, 3, 4, 5, 7, 10, 20 and 30 years. The CDS spreads are quoted on February 3, 2020. Companies are organized in three groups according to their S&P rating. The first group is made of 57 companies characterized by medium and high investment grade, that is credit rating greater than or equal to $A-$. The second group is made of 68 companies characterized by low investment grade, that is credit rating in the range $BBB+$ and $BBB-$. The third group is made of 17 companies characterized by speculative (no investment) grade, that is credit rating lower than $BBB-$. The statistics of the calibrations are available for each one of these three groups

**Table 4:** Statistics of the calibrated values for the credit risk model here proposed (formula $\Pi _{_{V}}$)

		$\frac{x_{0}}{x_{L}}$	$\alpha$	$\sigma _{x}$	*a*	*b*	$\lambda$	MAPE %
Finance and Insurance	Average	3.027	0.081	0.237	0.009	0.499	0.006	8.30%
	Min	1.015	$-$ 0.012	0.101	$-$ 0.020	$-$ 17.171	0.000	0.30%
	Max	4.574	1.304	0.300	0.010	1.747	0.099	15.80%
	SD	0.734	0.187	0.042	0.004	3.671	0.004	3.10%
Manufacturing	Average	2.990	0.038	0.234	0.010	1.577	0.001	8.20%
	Min	2.392	$-$ 0.012	0.177	0.010	0.003	0.000	4.90%
	Max	4.716	0.078	0.300	0.010	1.837	0.009	13.10%
	SD	0.435	0.019	0.025	0	0.363	0.001	2.10%
Information	Average	3.300	0.037	0.251	0.008	1.065	0.002	8.70%
	Min	2.636	0.005	0.198	$-$ 0.009	$-$ 3.881	0.000	1.50%
	Max	4.977	0.074	0.300	0.010	1.813	0.010	14.80%
	SD	0.674	0.021	0.032	0.006	1.580	0.002	3.10%
Utilities	Average	3.038	0.037	0.232	0.010	1.668	0.001	7.50%
	Min	2.472	0.013	0.192	0.010	1.494	0.000	4.60%
	Max	4.140	0.062	0.287	0.010	1.795	0.002	9.10%
	SD	0.474	0.013	0.027	0	0.000	0.101	1.50%
Transportation and Warehousing	Average	2.941	0.032	0.233	0.004	$-$ 3.231	0.020	7.20%
	Min	1.635	0.010	0.185	$-$ 0.020	$-$ 19.946	0.000	2.50%
	Max	4.936	0.063	0.300	0.010	1.860	0.100	19.20%
	SD	1.064	0.018	0.037	0.012	8.655	0.037	5.80%
Others	Average	3.033	0.027	0.243	0.008	0.748	0.004	7.10%
	Min	2.131	$-$ 0.030	0.200	$-$ 0.013	$-$ 3.881	0.000	1.50%
	Max	4.977	0.058	0.289	0.010	1.831	0.024	11.20%
	SD	0.636	0.022	0.027	0.006	1.750	0.006	2.40%

For each one of the 142 European companies listed in Appendix C, the parameters are calibrated by employing CDS spreads with maturities 6 months and 1, 2, 3, 4, 5, 7, 10, 20 and 30 years. The CDS spreads are quoted on February 3, 2020. Companies are organized in six groups according to the two digits 2017 NAICS sector code (see https://www.census.gov/naics/). The statistics of the calibrations are available for each one of these six groups

The results of this calibration are available in Table 3, where companies are classified into three groups according to their S&P rating. We observe that assuming the valuation framework in Sect. 2 we obtain a goodness of fit almost equal, and slightly higher, than the one obtained by assuming the credit risk model in Cathcart and El-Jahel (2003). This result is observable independently of the investment grade of the companies considered. Moreover, the values of the calibrated parameters are very similar between the credit risk model here proposed and the one in Cathcart and El-Jahel (2003). This indicates that the two models convey the same information about the credit quality of a firm. These findings are confirmed looking at the results of the calibrations for individual companies (see Appendix C, Table 7, where we report the calibration results for a selection of companies from different sectors, countries, and rating classes). Finally, Table 4 reports the calibration results aggregating the companies by industrial sector, and we see that the calibration exercise gives rather homogenous results across sectors. The values for the credit risk model in Cathcart and El-Jahel (2003) are not reported for brevity, although they do not show relevant differences from the valuation framework proposed in Sect. 2.

Then, we can conclude that considering a Vasicek process (as in the current valuation framework) instead of a CIR process (as in Cathcart and El-Jahel 2003) for modeling the short-term interest rate allows us to preserve (eventually improve) the goodness of fit, as well as to account for negative interest rates and to reduce the computational time required for the calibrations. As specified in Table 1, the gain in terms of CPU time is of about hundred times. Specifically, employing the proposed valuation framework, that is pricing formula (21), the time for calibrating a set of 142 term structure of CDS spreads is equal to 13 minutes and 22 seconds (802s), while employing the credit risk model in Cathcart and El-Jahel (2003), that is pricing formula (22), the same set of term structure of CDS spreads needs 20 hours, 11 minutes and 47 seconds (72707s) to be calibrated.

To bring the investigation into perspective, a comparison with the results in Ballestra et al. (2020) is required. In fact, the empirical testing conducted in Ballestra et al. (2020) considers: 1) The credit risk model proposed in Cathcart and El-Jahel (2003), which is the model here considered when the short-term interest rate follows a CIR process; 2) The advanced version of this model proposed in Cathcart and El-Jahel (2006), where the hazard rate also depends on the signaling process; 3) The credit risk model as the one here considered but with constant hazard rate; 4) The credit risk model as the one here considered but with zero hazard rate.

The results in Ballestra et al. (2020) underline that an evaluation framework as the one here considered but with constant hazard rate, i.e. *b* set equal to zero, represents a good compromise between goodness of fit and computational simplicity. Despite so, the same results indicate that the assumption of a constant hazard rate reduces the level of precision. On average, the reduction is estimated in a point of the mean absolute percentage error. This gap of goodness of fit is obtained by employing a set of 65 term structures of CDS spreads, which are made of only seven maturities, that is 1 year, 2 years, 3 years, 4 years, 5 years, 7 years and 10 years. Therefore, the gap is expected to be larger when, as in the current analysis, the further maturities of 6 months, 20 years and 30 years are considered.

These empirical findings compared to those obtained here lead us to a clear conclusion. The credit risk model here proposed matches the goodness of fit of the model in Cathcart and El-Jahel (2003) and, at the same time, ensures the same computational efficiency of the model with constant hazard rate.

All this confirms and enforces our conjecture. That is, assuming that the short-term interest rate follows a Vasicek process instead of a CIR process, we obtain a valid alternative to the credit risk model in Cathcart and El-Jahel (2003). Computational efficiency and high goodness of fit make the proposed model particularly useful for practitioners.

## Historical calibration of CDS curves

The credit risk model here considered is calibrated by using historical data. In particular, we employ the same set of 142 companies employed above but we consider the term structures of CDS spreads on three different dates, that is February 3, 2020, February 1, 2019 and February 1, 2018. The calibration procedure is analogous to the one used above. For each of these three dates, we obtain the maximum-likelihood estimation of the parameters of the Vasicek model using the monthly observations of the 1-week Euribor rate in the previous ten years. The current value of the default-free interest rate is set equal to the 1-week Euribor rate observed on the day the term structure of CDS spread is considered and the recovery rate is set to $40\%$. The remaining parameters are calibrated company by company by minimizing the mean absolute percentage error as in (23).

**Table 5:** Statistics of the calibrated values and of the mean absolute percentage error (goodness of fit) for the credit risk model here considered

		(AAA) – (A-)			(BBB+) – (BBB-)			(BB+) – (CCC)		
		2020	2019	2018	2020	2019	2018	2020	2019	2018
$\frac{x_{0}}{x_{L}}$	Average	3.224	3.225	3.428	2.999	3.009	3.180	2.583	2.560	2.904
	Min	2.392	1.842	1.813	1.612	1.600	1.667	1.015	1.014	1.511
	Max	4.716	4.465	4.999	4.977	4.327	4.518	4.936	3.126	3.972
	SD	0.588	0.604	0.738	0.558	0.513	0.638	0.831	0.503	0.651
$\alpha$	Average	0.054	0.052	0.048	0.037	0.035	0.040	0.084	0.071	0.017
	Min	0.022	0.007	0.018	0.009	0.004	$-0.032$	$-0.030$	$-0.082$	$-0.031$
	Max	0.121	0.121	0.121	0.188	0.087	0.094	1.304	1.286	0.071
	SD	0.020	0.020	0.019	0.023	0.016	0.020	0.315	0.315	0.030
$\sigma _{x}$	Average	0.237	0.240	0.230	0.236	0.246	0.239	0.244	0.249	0.253
	Min	0.177	0.125	0.121	0.101	0.100	0.103	0.114	0.111	0.102
	Max	0.300	0.300	0.298	0.300	0.300	0.297	0.300	0.291	0.300
	SD	0.032	0.036	0.036	0.030	0.035	0.042	0.047	0.049	0.063
*a*	Average	0.010	0.009	0.006	0.009	0.010	0.008	0.005	0.008	0.009
	Min	$-0.013$	0.004	0.001	$-0.009$	0.004	0.001	$-0.020$	$-0.020$	0.000
	Max	0.010	0.010	0.010	0.010	0.010	0.010	0.010	0.010	0.010
	SD	0.003	0.001	0.003	0.003	0.001	0.003	0.010	0.008	0.003
*b*	Average	1.506	2.059	1.407	1.277	1.487	1.552	$-3.434$	$-2.731$	$-1.324$
	Min	$-2.804$	0.098	0.257	$-3.881$	$-6.739$	$-3.552$	$-19.946$	$-32.061$	$-51.891$
	Max	1.860	2.618	2.456	1.822	2.641	2.615	1.638	2.261	2.473
	SD	0.672	0.539	0.659	1.075	1.801	0.922	7.351	8.648	13.053
$\lambda$	Average	0.002	0.002	0.001	0.003	0.004	0.002	0.023	0.018	0.014
	Min	0.000	0.000	0.000	0.001	0.000	0.000	0.001	0.001	0.000
	Max	0.010	0.010	0.009	0.022	0.035	0.023	0.100	0.129	0.200
	SD	0.002	0.002	0.002	0.003	0.007	0.003	0.033	0.032	0.048
MAPE$\%$	Average	8.520	4.795	3.862	8.058	5.071	4.875	5.700	5.769	5.406
	Min	2.253	1.698	1.319	0.305	1.647	1.870	1.863	1.244	2.117
	Max	19.234	9.673	14.753	15.765	11.842	19.870	10.188	9.232	8.565
	SD	2.507	1.771	2.374	2.920	1.879	3.220	2.393	2.254	1.818

For each one of the 142 European companies listed in Appendix C, the parameters are calibrated by employing CDS spreads with maturities 6 months and 1, 2, 3, 4, 5, 7, 10, 20 and 30 years. The CDS spreads used for the calibrations are quoted on February 3, 2020, on February 1, 2019, and on February 1, 2018. Companies are organized in three groups according to their S&P rating. The first group is made of 57 companies characterized by medium and high investment grade, that is credit rating greater than or equal to $A-$. The second group is made of 68 companies characterized by low investment grade, that is credit rating in the range $BBB+$ and $BBB-$. The third group is made of 17 companies characterized by speculative (no investment) grade, that is credit rating lower than $BBB-$. The statistics of the calibrations are available for each one of these three groups

The statistics of the calibrations are available in Table 5. They indicate the stability of the values of the calibrated parameters over time. In fact, for each calibrated parameter we observe similar average value, minimum value, maximum value and standard division. The only exception is the parameter $\alpha$ for non-investment grade companies ((BB+)-(CCC)), where we observe that the average value of $\alpha$ is 0.071 in 2019 while it jumps to 0.017 in 2018. However, this difference is due to a single company with credit spreads of around 1500 basis points for short maturities (an outlier), that is a company that data indicate in strong financial distress. The details of these calibrations are available in Appendix C, see Table 8, where the estimated parameters are available for a selection of companies.

The statistics of the calibrations available in Table 5 also provide interesting insights about the financial situations of companies with different credit rating. Considering the parameter $\frac{x_{0}}{x_{L}}$, its value increases with the company’s credit rating. This is consistent with the fact that the credit quality of a company with a high credit rating should be higher than the one of a company with a low credit rating. The volatility of the signaling variable increases reducing the credit rating of the firm. This indicates that the creditworthiness (or credit quality) of companies with a low credit rating is more sensitive to random shocks (more subject to fluctuations).^6^ At the same time, the hazard rate increases on average more than ten times moving from companies with high credit rating, that is (AAA)-(A-), to companies with low credit rating, that is (BB+)-(CCC). This indicates that the probability of an unexpected default is higher for firms with a low credit rating. Moreover, we observe that the value of the parameter *b* is on average positive for companies with credit rating higher than or equal to BBB-. Therefore these companies are characterized by a positive duration gap. On the contrary, companies with a credit rating lower than BBB- are characterized by a negative value of *b*. Therefore these companies are characterized by a negative duration gap.

The negative duration gap for companies with low credit ratings indicates that these companies are more financially exposed than companies with high credit ratings. Moreover, for a company with positive (negative) duration gap the value of the activities increases more (less) than the values of the liabilities when the interest rate decreases. Thus, a reduction in risk-free interest rates makes low-credit-rated companies even more fragile, while high-credit-rated companies benefit from the reduction. As suggested by the hazard rate of the credit risk models considered in this work.^7^

**Fig. 5:**
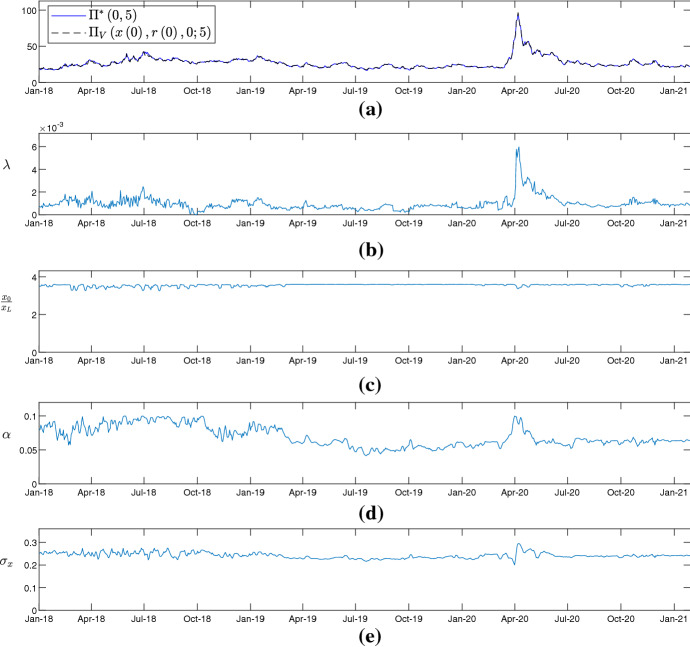
Rolling window calibrations for the CDS pricing formula (21) using daily observations of the CDS spreads written on debt issued by Allianz SE. Time window from 1-1-2018 to 29-1-2021. Panel (**a**), empirical 5-year CDS spreads (blue line) and theoretical 5-year CDS spreads (black and dashed line). Panel (**b**), calibrated values of the intensity of default $\left( \lambda \right)$. Panel (**c**), calibrated values of the ration between signaling variable and default barrier $\left( \frac{x_{0}}{x_{L}}\right)$. Panel (**d**), calibrated values of the drift parameter of the signaling variable $\left( \alpha \right)$. Panel (**e**), calibrated values of the short-term volatility of the signaling variable $\left( \sigma _{x}\right)$

**Fig. 6:**
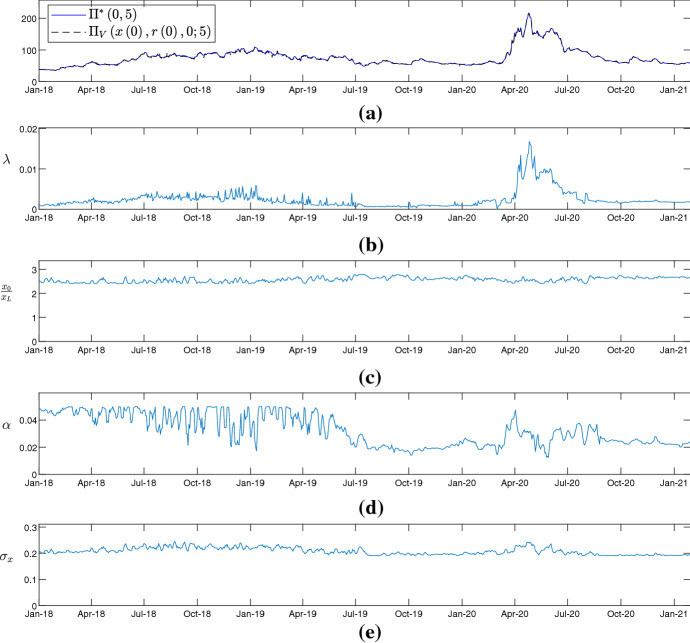
Rolling window calibrations for the CDS pricing formula (21) using daily observations of the CDS spreads written on debt issued by Dailmer AG. Time window from 1-1-2018 to 29-1-2021. **a** empirical 5-year CDS spreads (blue line) and theoretical 5-year CDS spreads (black and dashed line). **b** calibrated values of the intensity of default $\left( \lambda \right)$. **c** calibrated values of the ration between signaling variable and default barrier $\left( \frac{x_{0}}{x_{L}}\right)$. **d** calibrated values of the drift parameter of the signaling variable $\left( \alpha \right)$. **e** calibrated values of the short-term volatility of the signaling variable $\left( \sigma _{x}\right)$

Finally, focusing on two companies, that is Allianz Se and Dailmer AG, the credit risk model is calibrated every trading day in the period from January 1, 2018 to January 29, 2021. For each trading day, the calibration procedure employed is the one described at the beginning of this section. The results of the calibrations are shown in Figs. 5 and 6 and indicate how the valuation framework proposed captures the impact of special events such as the COVID-19 pandemic. Specifically, the parameters of the credit-quality signaling variable remain relatively stable over time. On the contrary, the intensity of default increases substantially in conjunction with COVID-19 pandemic. This allows to replicate the inflated CDS spreads that are observed in the period from March to April, 2020. In these two months the 5-year CDS spreads almost quadruple. The model therefore captures that the increase in CDS spreads is not due to firm-specific components but is due to macroeconomic events or endogenous shocks, specifically the COVID-19 pandemic.

## Conclusions

In this work, a revised version of the credit risk model proposed in Cathcart and El-Jahel (2003) is considered. Specifically, the short-term interest rate is assumed to follow a Vasicek process instead of a CIR process. This allows us to account for negative interest rates, which are empirically observed in the last decade. Then, a formula for pricing CDS spreads is derived, which requires a hundred times less CPU time than the CDS pricing formula for the credit risk model in Cathcart and El-Jahel (2003). In addition, an empirical analysis reveals that these two pricing formulas ensure the same goodness of fit. Therefore, the credit risk model proposed represents a valid alternative that may be of interest for practitioners. The validity of the model is also corroborated by the values of the parameters which, calibrated using the term structures of CDS spreads for 142 European companies, show large stability over time. Further applications of the model could focus on the study of the potential transmission of credit risk across sovereign, bank, and non-bank institutions (see e.g. Gross and Siklos 2020), or in the modeling of quanto CDS spreads (i.e. differences in CDS premia in different currency denominations, see e.g. Della Corte et al. 2018). We leave these streams of research to further works.

## Appendix A

Assuming that the risk-free rate follows a CIR process instead of a Vasicek process, the valuation framework in Sect. 2 becomes the hybrid credit risk model proposed in Cathcart and El-Jahel (2003).

Specifically, Cathcart and El-Jahel (2003) assumes that the risk-free rate follows process (1) where $\sigma _{r}\left( t,r\left( t\right) \right) =\sigma _{r}\sqrt{r\left( t\right) }$. In this case, the risk-neutral price at the current time $t_{0}$ of a default-free pure discount bond with maturity *T* is given by (see again Cox et al. 1985):

24 $$\begin{aligned} P_{_{CIR}}\left( r\left( t_{0}\right) ,t_{0};T\right) =e^{\displaystyle C\left( t_{0};T\right) +D\left( t_{0};T\right) r\left( t_{0}\right) } \end{aligned}$$

where

25 $$\begin{aligned} C\left( t_{0};T\right)= & {} \frac{2k\mu }{\sigma _{r}^{2}}\ln \left( \frac{2\varphi e^{\frac{\left( k+\varphi \right) \left( T-t_{0}\right) }{2}}}{2\varphi +\left( k+\varphi \right) \left( e^{\varphi \left( T-t_{0}\right) }-1\right) }\right) \nonumber \\ D\left( t_{0};T\right)= & {} \frac{-2\left( e^{\varphi \left( T-t_{0}\right) }-1\right) }{2\varphi +\left( k+\varphi \right) \left( e^{\varphi \left( T-t_{0}\right) }-1\right) } \end{aligned}$$

and $$\varphi =\sqrt{k^2+2\sigma _{r}^{2}}$$.

Considering the credit risk model in Cathcart and El-Jahel (2003), the formula for the probability to survive as well as the formula for the current price of a survival security are derived in the following Proposition (proof in Appendix B).

### Proposition 5

Assuming the valuation framework in Cathcart and El-Jahel (2003), we have that the probability to survive up to time *T* is given by

26 $$\begin{aligned} Q\left( t_{d}>T\right) = f\left( x\left( t_{0}\right) ,t_{0};T\right) g_{_{CIR}}\left( r\left( t_{0}\right) ,t_{0};T\right) \end{aligned}$$

where *f* is defined in (6), while

27 $$\begin{aligned} g_{_{CIR}}\left( r\left( t_{0}\right) ,t_{0};T\right) =e^{\displaystyle E_{5}\left( t_{0};T\right) +E_{6}\left( t_{0};T\right) r\left( t_{0}\right) } \end{aligned}$$

where

28 $$\begin{aligned} E_{5}\left( t_{0};T\right)= & {} -a\left( T-t_{0}\right) + \frac{2k\mu }{\sigma _{r}^2 }\ln \left( \frac{2\gamma e^{ \frac{\left( k+\gamma \right) \left( T-t_{0}\right) }{2} }}{2\gamma +\left( k+\gamma \right) \left( e^{\gamma \left( T-t_{0}\right) } -1\right) }\right) \nonumber \\ E_{6}\left( t_{0};T\right)= & {} -\frac{2b\left( e^{\gamma \left( T-t_{0}\right) }-1\right) }{2\gamma +\left( k+\gamma \right) \left( e^{\gamma \left( T-t_{0}\right) } -1\right) } \end{aligned}$$

and $\gamma = \sqrt{k^2+2b\sigma _{r}^{2}}$. The current price of a survival security with unitary face value and maturity *T* is given by

29 $$\begin{aligned} S_{_{CIR}}\left( r\left( t_{0}\right) ,x\left( t_{0}\right) ,t_{0};T\right) = f\left( x\left( t_{0}\right) ,t_{0};T\right) \tilde{g}_{_{CIR}}\left( r\left( t_{0}\right) ,t_{0};T\right) \end{aligned}$$

where $\tilde{g}_{_{CIR}}$ is obtained by $g_{_{CIR}}$ substituting *b* with $b+1$.

The current price of a survival security in Proposition 5 is the discounted value of the probability to survive when the short-term interest rate follows a CIR process. The formula for the current price of a survival security is available in closed form as shown in Proposition 5. Conversely, the forward price of a survival security does not admit a closed-form solution when the credit risk model in Cathcart and El-Jahel (2003) is employed.

Consider the *forward measure* $Q^{T}$ to compute the forward price of a survival security, see, e.g., Madan and Unal (2000). We recap that $Q^{T}$ is the unique equivalent martingale measure under which the values of traded assets discounted by the price of the default-free bond, in this case $P_{_{CIR}}\left( r\left( t_{0}\right) ,t_{0};T\right)$, are martingales.

The change of measure density process from *Q* to $Q^{T}$ is given by the Radon-Nikodym derivative which is equal to the price of a risk-free bond with maturity *T*, see, e.g., Brigo and Mercurio (2007) and Madan and Unal (2000). Then, applying Girsanov’s theorem, the $Q^{T}$ dynamics for *x* and *r* are given by

30 $$\begin{aligned} \text {d}x\left( t\right)= & {} \alpha x\left( t\right) \text {d}t+\sigma _{x} \text {d}W_{x}\left( t\right) \nonumber \\ \text {d}r\left( t\right)= & {} \left( \mu \left( k-r\left( t\right) \right) - \sigma _{x}^{2} D\left( t;T\right) r\left( t\right) \right) \text {d}t + \sigma _{r} d\tilde{W}_{r}\left( t\right) \end{aligned}$$

where $W_{x}\left( t\right)$ and $\tilde{W}_{r}\left( t\right)$ are Wiener processes under $Q^{T}$ while $D\left( t;T\right)$ is defined in (25). Since by assumption the signaling variable is uncorrelated to the short-term interest rate, its dynamics does not change when we move from the martingale measure *Q* to the forward measure $Q^{T}$, i.e. $Q^{T}\left( \tau _{x}>T\right) =Q\left( \tau _{x}>T\right)$.

### Proposition 6

Assuming the valuation framework in Cathcart and El-Jahel (2003), the forward price of a survival security with unitary face value and maturity *T* is given by

31 $$\begin{aligned} F_{_{CIR}}\left( x\left( t_{0}\right) ,r\left( t_{0}\right) ,t_{0};T\right)= & {} Q^{T}\left( t_{d}>T\right) \nonumber \\= & {} f\left( x\left( t_{0}\right) ,t_{0};T\right) h_{_{CIR}}\left( r\left( t_{0}\right) ,t_{0};T\right) \end{aligned}$$

where *f* is defined in (6), while

32 $$\begin{aligned} h_{_{CIR}}\left( r\left( t_{0}\right) ,t_{0};T\right) = e^{\displaystyle E_{7}\left( t_{0};T\right) +E_{8}\left( t_{0};T\right) r\left( t_{0}\right) } \end{aligned}$$

with $E_{7}$ and $E_{8}$ that solve the following system of two ordinary differential equations

33 $$\begin{aligned} E_{8,t}= & {} k E_{8} +b-\frac{\sigma _{r}^{2}}{2}E_{8}^{2} -\sigma _{r}^{2}D\left( t,T\right) E_{8}\nonumber \\ E_{7,t}= & {} - k\mu E_{8}+a \end{aligned}$$

subject to final conditions $E_{7}\left( T,T\right) = E_{8}\left( T,T\right) =0$. $D\left( \cdot ;\cdot \right)$ is defined as in (25).

Note that in order to compute the forward-price of a survival security, system (33) needs to be approximated numerically.

## Appendix B

### Proof of Proposition 1

Employing the results in (5)–(8), to derive the probability to survive $Q\left( t_{d}>T\right)$ we only need to compute $\mathbb {E}^{Q}\left[ \mathbf {1}_{\left\{ \tau _{r}>T\right\} } \right]$. By standard results on Cox processes, we have that

34 $$\begin{aligned} g_{_{V}}\left( r\left( t\right) ,t;T\right)= & {} \mathbb {E}^{Q}\left[ \mathbf {1}_{\left\{ \tau _{r}>T\right\} } \right] \nonumber \\= & {} \mathbb {E}^{Q}\left[ e^{-\int _{t}^{T}\lambda \left( r\left( s\right) \right) ds}\right] \end{aligned}$$

where $\lambda \left( r\right) =a+b r$ is the intensity of default. Since by assumption *r* follows (under martingale measure *Q*) the Vasicek process (1), by Feynman-Kac formula, see, e.g., Friedman (1975) and Krylov (1980), we know that the function *g* satisfies the following partial differential problem:

35 $$\begin{aligned} -g_{_{V},t}-k\left( \mu -r\right) g_{_{V},r}-\frac{\sigma _{r}^{2}}{2}g_{_{V},rr}+\lambda \left( r\right) g_{_{V}}=0 \end{aligned}$$

subject to

36 $$\begin{aligned} g_{_{V}}\left( r\left( T\right) ,T;T\right) =1 \end{aligned}$$

Guessing a solution of the form

37 $$\begin{aligned} g_{_{V}}\left( r\left( t\right) ,t;T\right) = e^{\displaystyle E_{1}\left( t;T\right) +E_{2}\left( t;T\right) r\left( t\right) } \end{aligned}$$

we obtain

38 $$\begin{aligned} -E_{1,t}-E_{2,t}r-k\left( \mu -r\right) E_{2}-\frac{\sigma _{r}^{2}}{2}E_{2}^{2}+a+br =0 \end{aligned}$$

Then, by separation of variables we have the following system of two ordinary differential equations^8^

39 $$\begin{aligned} E_{2,t}= & {} kE_{2} + b\nonumber \\ E_{1,t}= & {} -k \mu E_{2}-\frac{\sigma _{r}^{2}}{2}E_{2}^{2}+a \end{aligned}$$

from which, imposing the condition $E_{2}\left( T;T\right) =0$, we obtain

40 $$\begin{aligned} E_{2}\left( t;T\right) = b\frac{e^{-k\left( T-t\right) }-1}{k} \end{aligned}$$

It follows that

41 $$\begin{aligned} E_{1}\left( t;T\right)= & {} \displaystyle \int _{0}^{t}\left( -k \mu E_{2}-\frac{\sigma _{r}^{2}}{2}E_{2}^{2}+a\right) ds + C_{0}\nonumber \\= & {} -\mu b\frac{e^{-k\left( T-t\right) }-e^{-kT}}{k}+ \mu b t -\frac{b^2 \sigma _{r}^{2}}{4k^3}e^{-2kT}\left( e^{2kt}-1\right) - \frac{b^2 \sigma _{r}^{2}}{2 k^2}t\nonumber \\&+\frac{2\sigma _{r}^{2} b^2}{2k^3}\left( e^{-k\left( T-t\right) }-e^{-kT}\right) +at+C_{0} \end{aligned}$$

Imposing $E_{1}\left( T;T\right) =0$, we obtain

42 $$\begin{aligned} E_{1}\left( t;T\right) = \left( \frac{b \sigma _{r}^{2} }{ 2k^2}-\mu \right) \left( E_{1}\left( t;T\right) + b\left( T-t\right) \right) -\frac{ \sigma _{r}^{2}}{4k}E^{2}_{1}\left( t;T\right) -a\left( T-t\right) \end{aligned}$$

By definition, the price of a survival security with unitary face value is given by

43 $$\begin{aligned} \mathbb {E}^{Q}\left[ \mathbf {1}_{\left\{ t_{d}>T\right\} }e^{-\int _{t}^{T}r\left( s\right) ds}\right] \end{aligned}$$

Since *x* and *r* are independent, (43) can be rewritten as

44 $$\begin{aligned} \mathbb {E}^{Q}\left[ \mathbf {1}_{\left\{ \tau _{x}>T\right\} }\right] \mathbb {E}^{Q}\left[ \mathbf {1}_{\left\{ \tau _{r}>T\right\} }e^{-\int _{t}^{T}r\left( s\right) ds}\right] \end{aligned}$$

where $\mathbb {E}^{Q}\left[ \mathbf {1}_{\left\{ \tau _{x}>T\right\} }\right]$ is defined as in (6)–(8), while by standard results on Cox processes, we have that

45 $$\begin{aligned} \mathbb {E}^{Q}\left[ \mathbf {1}_{\left\{ \tau _{r}>T\right\} }e^{-\int _{t}^{T}r\left( s\right) ds}\right] =\mathbb {E}^{Q}\left[ e^{-\int _{t}^{T}\left( \lambda \left( r\left( s\right) \right) +r\left( s\right) \right) ds}\right] \end{aligned}$$

Therefore, this expected value is equal to $\tilde{g}_{_{V}}$, which is obtained by $g_{_{V}}$ substituting *b* with $b+1$. This completes the proof. $\square$

### Proof of Proposition 2

Note that $Q^{T}\left( t_{d}>T\right) =\mathbb {E}^{Q^{T}}\left[ \mathbf {1}_{\left\{ \tau _{x}>T\right\} }\right] \mathbb {E}^{Q^{T}}\left[ \mathbf {1}_{\left\{ \tau _{r}>T\right\} } \right]$ and $\mathbb {E}^{Q^{T}}\left[ \mathbf {1}_{\left\{ \tau _{x}>T\right\} }\right] = \mathbb {E}^{Q}\left[ \mathbf {1}_{\left\{ \tau _{x}>T\right\} }\right]$ which is equal to *f* as shown in (6)–(8). Therefore, we need only to compute $\mathbb {E}^{Q^{T}}\left[ \mathbf {1}_{\left\{ \tau _{r}>T\right\} } \right]$. By standard results on Cox processes, we have that

46 $$\begin{aligned} h_{_{V}}\left( r\left( t\right) ,t;T\right)= & {} \mathbb {E}^{Q^{T}}\left[ \mathbf {1}_{\left\{ \tau _{r}>T\right\} } \right] \nonumber \\= & {} \mathbb {E}^{Q^{T}}\left[ e^{-\int _{t}^{T}\lambda \left( r\left( s\right) \right) ds}\right] \end{aligned}$$

where $\lambda \left( r\right) =a+b r$. Since *r* follows process (30) under forward measure $Q^{T}$, by Feynman-Kac formula, see, e.g., Friedman (1975) and Krylov (1980), we know that the function $h_{V}$ satisfies the following partial differential problem:

47 $$\begin{aligned} -h_{_{V},t}-\left( k\left( \mu -r\right) - \sigma _{r}^{2}B\left( t,T\right) \right) h_{_{V},r}-\frac{\sigma _{r}^{2}}{2}h_{_{V},rr}+\lambda \left( r\right) h_{_{V}}=0 \end{aligned}$$

subject to

48 $$\begin{aligned} h_{_{V}}\left( r\left( T\right) ,T;T\right) =1 \end{aligned}$$

where $B\left( t,T\right)$ is defined as in (3). Guessing a solution of the form

49 $$\begin{aligned} h_{_{V}}\left( r\left( t\right) ,t;T\right) = e^{\displaystyle E_{3}\left( t,T\right) +E_{4}\left( t,T\right) r\left( t\right) } \end{aligned}$$

we have that

50 $$\begin{aligned} -\left( E_{3,t}+E_{4,t}r\right) -\left( k\left( \mu -r\right) - \sigma _{r}^{2}B\left( t,T\right) \right) E_{4}-\frac{\sigma _{r}^{2}}{2}E_{4}^{2}+\lambda \left( r\right) =0 \end{aligned}$$

By separation of variables, we obtain

51 $$\begin{aligned} E_{4,t}= & {} k E_{4} +b \nonumber \\ E_{3,t}= & {} -\left( k\mu - \sigma _{r}^{2}B\left( t,T\right) \right) E_{4}-\frac{\sigma _{r}^{2}}{2}E_{4}^{2}+a \end{aligned}$$

Therefore, $$E_{4}\left( t;T\right) =bB\left( t;T\right) $$ and

52 $$\begin{aligned} E_{3}\left( t;T\right)&= \displaystyle \int _{T}^{t}\left( - k\mu E_{4}\left( s;T\right) + \sigma _{r}^{2}B\left( s,T\right) E_{4}\left( s;T\right) \right) ds\nonumber \\&+\displaystyle \int _{T}^{t}\left( -\frac{\sigma _{r}^{2}}{2}E_{4}^{2}\left( s;T\right) +a\right) ds \end{aligned}$$

Evaluating the integral we obtain $E_{3}\left( t;T\right)$ as in (16). This completes the proof. $\square$

### Proof of Proposition 3

Consider the forward measure $Q^{T}$. Then the forward price of the protection leg is given by

53 $$\begin{aligned} E^{Q^{T}}\left[ \left( 1-\delta \right) \mathbf {1}_{\left\{ T>t_{d}>t_{0}\right\} }\right]= & {} \left( 1-\delta \right) E^{Q^{T}}\left[ 1-\mathbf {1}_{\left\{ \tau _{x}>T\right\} } \mathbf {1}_{\left\{ \tau _{r}>T\right\} }\right] \nonumber \\= & {} \left( 1-\delta \right) \left( 1-E^{Q^{T}}\left[ \mathbf {1}_{\left\{ \tau _{x}>T\right\} }\right] E^{Q^{T}}\left[ \mathbf {1}_{\left\{ \tau _{r}>T\right\} }\right] \right) \end{aligned}$$

where $E^{Q^{T}}\left[ \mathbf {1}_{\left\{ \tau _{x}>T\right\} }\right] E^{Q^{T}}\left[ \mathbf {1}_{\left\{ \tau _{r}>T\right\} }\right]$ is by definition the probability to survive under the forward measure (or forward price of a survival security). Since *r* follows process (30) under forward measure $Q^{T}$, the formula for the forward price of a survival security is $F_{_{V}}\left( x,r,t;T\right)$ defined as in (14), see Proposition 2. Therefore, the current price $\Phi$ of the protection leg is given by:

54 $$\begin{aligned} \Phi \left( x,r,t;T\right) =P_{V}\left( r,t;T\right) \left( 1-F_{_{V}}\left( x,r,t;T\right) \right) \left( 1-\delta \right) \end{aligned}$$

where $P_{V}\left( r,t;T\right)$ is the current price of a default-free pure discount bond defined as in (2). Concerning the premium leg, by Fubini’s theorem we have that

55 $$\begin{aligned} \mathbb {E}^{Q}\left[ \int _{t_{0}}^{T}\mathbf {1}_{\left\{ \tau _{x}>z\wedge \tau _{r}>z\right\} } e^{-\int _{t_{0}}^{z}r\left( s\right) ds}dz\right] =\int _{t_{0}}^{T}\mathbb {E}^{Q}\left[ \mathbf {1}_{\left\{ \tau _{x}>z\wedge \tau _{r}>z\right\} } e^{-\int _{t_{0}}^{z}r\left( s\right) ds}\right] dz \end{aligned}$$

where $\mathbb {E}^{Q}\left[ \mathbf {1}_{\left\{ \tau _{x}>z\wedge \tau _{r}>z\right\} }e^{-\int _{t_{0}}^{z}r\left( s\right) ds}\right]$ is, by definition, the current price of a survival security with unitary face value. Since by assumption *r* follows (under martingale measure *Q*) the Vasicek process (1), the formula for the current price of a survival security is given in (12), see Proposition 1. This completes the proof of the Proposition. $\square$

### Proof of Proposition 5

Employing the results in (5)–(8), we need only to compute $\mathbb {E}^{Q}\left[ \mathbf {1}_{\left\{ \tau _{r}>T\right\} } \right]$ in order to derive the probability to survive $Q\left( t_{d}>T\right)$. By standard results on Cox processes, we have that

56 $$\begin{aligned} g_{_{CIR}}\left( r\left( t\right) ,t;T\right)= & {} \mathbb {E}^{Q}\left[ \mathbf {1}_{\left\{ \tau _{r}>T\right\} } \right] \nonumber \\= & {} \mathbb {E}^{Q}\left[ e^{-\int _{t}^{T}\lambda \left( r\left( s\right) \right) ds}\right] \end{aligned}$$

where $\lambda \left( r\right) =a+b r$ is the intensity of default. Since by assumption *r* follows (under martingale measure *Q*) the CIR process as indicated in Appendix A, by Feynman-Kac formula, see, e.g., Friedman (1975) and Krylov (1980), we know that the function $g_{_{CIR}}$ satisfies the following partial differential problem:

57 $$\begin{aligned} -g_{_{CIR},t}-k\left( \mu -r\right) g_{_{CIR},r}-\frac{\sigma _{r}^{2}}{2}rg_{_{CIR},rr}+\lambda \left( r\right) g_{_{CIR}}=0 \end{aligned}$$

subject to

58 $$\begin{aligned} g_{_{CIR}}\left( r\left( T\right) ,T;T\right) =1 \end{aligned}$$

Guessing a solution of the form

59 $$\begin{aligned} g_{_{CIR}}\left( r,t;T\right) =e^{\displaystyle E_{5}\left( t;T\right) +E_{6}\left( t;T\right) r\left( t\right) } \end{aligned}$$

we obtain

60 $$\begin{aligned} -E_{5,t}-E_{6,t}r-k\left( \mu -r\right) E_{6}-\frac{\sigma _{r}^{2}}{2}rE_{6}^{2}+a+br=0 \end{aligned}$$

Then, by separation of variables we have the following system of two ordinary differential equations:

61 $$\begin{aligned} E_{5,t}= & {} a - k \mu E_{6} \nonumber \\ E_{6,t}= & {} b+kE_{6}-\frac{\sigma _{r}^{2}}{2}E_{6}^{2} \end{aligned}$$

where the second one is a Riccati equation, subject to the conditions $E_{5}\left( T;T\right) =E_{6}\left( T;T\right) =0$. Following (Polyanin and Zaitsev 2003, sec.1.2.1), set

62 $$\begin{aligned} \frac{\sigma _{r}^{2}}{2} E_{6}\left( t;T\right) =\frac{u_{t}\left( t;T\right) }{u\left( t;T\right) } \end{aligned}$$

from which we have that

63 $$\begin{aligned} E_{6,t} = \frac{2}{\sigma _{r}^{2}} \frac{u_{tt}u-\left( u_{t}\right) ^2}{u^2} = b+k\frac{2}{\sigma _{r}^{2}}\frac{u_{t}}{u} -\frac{2}{\sigma _{r}^{2}}\frac{\left( u_{t}\right) ^2}{u^2} \end{aligned}$$

Therefore, *u* satisfies the following second-order ordinary differential equation

64 $$\begin{aligned} u_{tt} - k u_{t} - b\frac{\sigma _{r}^{2}}{2}u = 0 \end{aligned}$$

Since $\gamma ^2 = k^2+2 b \sigma _{r}^{2} >0$, its solution, see (Polyanin and Zaitsev 2003, sec. 2.1.2-2), is given by

65 $$\begin{aligned} u =C_{1}e^{\frac{1}{2}\left( k+\gamma \right) t}+C_{2}e^{\frac{1}{2}\left( k-\gamma \right) t} \end{aligned}$$

and its derivative is

66 $$\begin{aligned} u_{t} =C_{1}\frac{1}{2}\left( k+\gamma \right) e^{\frac{1}{2}\left( k+\gamma \right) t}+C_{2}\frac{1}{2}\left( k-\gamma \right) e^{\frac{1}{2}\left( k-\gamma \right) t} \end{aligned}$$

By imposing $u_{t}=0$ in $t=T$, which allow us to have the final condition $E_{6}\left( T;T\right) =0$ satisfied, we obtain

67 $$\begin{aligned} C_{2} = - C_{1}\frac{k+\gamma }{k-\gamma }e^{\gamma T} \end{aligned}$$

Therefore,

68 $$\begin{aligned} u =C_{1} e^{\frac{1}{2}\left( k+\gamma \right) t} - C_{1}\frac{k+\gamma }{k-\gamma }e^{\gamma T} e^{+\frac{1}{2}\left( k-\gamma \right) t} \end{aligned}$$

and

69 $$\begin{aligned} E_{6}\left( t;T\right)= & {} \frac{2}{\sigma _{r}^{2}}\frac{C_{1}\frac{1}{2}\left( k+\gamma \right) e^{\frac{1}{2}\left( k+\gamma \right) t}- C_{1}e^{\gamma T}\frac{1}{2}\left( k+\gamma \right) e^{\frac{1}{2}\left( k-\gamma \right) t}}{C_{1} e^{\frac{1}{2}\left( k+\gamma \right) t} - C_{1}\frac{k+\gamma }{k-\gamma }e^{\gamma T} e^{+\frac{1}{2}\left( k-\gamma \right) t}}\nonumber \\= & {} -\frac{2b\left( e^{\gamma \left( T-t\right) }-1\right) }{2\gamma +\left( k+\gamma \right) \left( e^{\gamma \left( T-t\right) } -1\right) } \end{aligned}$$

Then,

70 $$\begin{aligned} E_{5}\left( t;T\right)= & {} \displaystyle \int _{T}^{t}ads - k\mu \int _{T}^{t} E_{6}\left( s;T\right) ds\nonumber \\= & {} - a\left( T-t\right) + \frac{2k\mu }{\sigma _{r}^2 }\ln \frac{2\gamma e^{ \frac{\left( k+\gamma \right) }{2} \left( T-t\right) }}{2\gamma +\left( k+\gamma \right) \left( e^{\gamma \left( T-t\right) } -1\right) } \end{aligned}$$

By definition, the price of a survival security with unitary face value is given by

71 $$\begin{aligned} \mathbb {E}^{Q}\left[ \mathbf {1}_{\left\{ t_{d}>T\right\} }e^{-\int _{t}^{T}r\left( s\right) ds}\right] \end{aligned}$$

Since *x* and *r* are independent, (71) can be rewritten as

72 $$\begin{aligned} \mathbb {E}^{Q}\left[ \mathbf {1}_{\left\{ \tau _{x}>T\right\} }\right] \mathbb {E}^{Q}\left[ \mathbf {1}_{\left\{ \tau _{r}>T\right\} }e^{-\int _{t}^{T}\lambda \left( r\left( s\right) \right) ds}\right] \end{aligned}$$

where $\mathbb {E}^{Q}\left[ \mathbf {1}_{\left\{ \tau _{x}>T\right\} }\right]$ is defined as in (6)–(8), while by standard results on Cox processes, we have that

73 $$\begin{aligned} \mathbb {E}^{Q}\left[ \mathbf {1}_{\left\{ \tau _{r}>T\right\} }e^{-\int _{t}^{T}r\left( s\right) ds}\right] =\mathbb {E}^{Q}\left[ e^{-\int _{t}^{T}\left( \lambda \left( r\left( s\right) \right) +r\left( s\right) \right) ds}\right] \end{aligned}$$

Therefore, this expected value is equal to $\tilde{g}_{_{CIR}}$, which is obtained by $g_{_{CIR}}$ substituting *b* with $b+1$. This completes the proof. $\square$

### Proof of Proposition 6

Note that $Q^{T}\left( t_{d}>T\right) =\mathbb {E}^{Q^{T}}\left[ \mathbf {1}_{\left\{ \tau _{x}>T\right\} }\right] \mathbb {E}^{Q^{T}}\left[ \mathbf {1}_{\left\{ \tau _{r}>T\right\} } \right]$ and $\mathbb {E}^{Q^{T}}\left[ \mathbf {1}_{\left\{ \tau _{x}>T\right\} }\right] = \mathbb {E}^{Q}\left[ \mathbf {1}_{\left\{ \tau _{x}>T\right\} }\right]$ which is equal to *f* as shown in (6)–(8). Therefore, we need only to compute $\mathbb {E}^{Q^{T}}\left[ \mathbf {1}_{\left\{ \tau _{r}>T\right\} } \right]$. By standard results on Cox processes, we have that

74 $$\begin{aligned} h_{_{CIR}}\left( r\left( t\right) ,t;T\right)= & {} \mathbb {E}^{Q^{T}}\left[ \mathbf {1}_{\left\{ \tau _{r}>T\right\} } \right] \nonumber \\= & {} \mathbb {E}^{Q^{T}}\left[ e^{-\int _{t}^{T}\lambda \left( r\left( s\right) \right) ds}\right] \end{aligned}$$

where $\lambda \left( r\right) =a+b r$ is the intensity of default. Since *r* follows process (30) under forward measure $Q^{T}$, by Feynman-Kac formula, see, e.g., Friedman (1975) and Krylov (1980), we know that the function $h_{_{CIR}}$ satisfies the following partial differential problem:

75 $$\begin{aligned} -h_{_{CIR},t}-\left( k\left( \mu -r\right) - \sigma _{r}^{2}D\left( t,T\right) r\right) h_{_{CIR},r}-\frac{\sigma _{r}^{2}}{2}rh_{_{CIR},rr}+\lambda \left( r\right) h_{_{CIR}}=0 \end{aligned}$$

subject to

76 $$\begin{aligned} h_{_{CIR}}\left( r\left( T\right) ,T;T\right) =1 \end{aligned}$$

where $D\left( t,T\right) =\frac{-2\left( 1-\exp \left( -\varphi \left( T-t_{0}\right) \right) \right) }{2\varphi +\left( k-\varphi \right) \exp \left( -\varphi \left( T-t_{0}\right) \right) }$ is as in (25). Guessing a solution of the form

77 $$\begin{aligned} h_{_{CIR}}\left( r\left( t\right) ,t;T\right) = e^{\displaystyle E_{7}\left( t,T\right) +E_{8}\left( t,T\right) r\left( t\right) } \end{aligned}$$

we obtain

78 $$\begin{aligned} -\left( E_{7,t}+E_{8,t}r\right) -\left( k\left( \mu -r\right) - \sigma _{r}^{2}D\left( t,T\right) r\right) E_{8}-\frac{\sigma _{r}^{2}}{2}rE_{8}^{2}+\lambda \left( r\right) =0 \end{aligned}$$

By separation of variables, we have the system of ordinary differential equations in (33) subject to final condition $E_{7}\left( T,T\right) = E_{8}\left( T,T\right) =0$. This completes the proof. $\square$

**Table 6:** Statistics of the CDS spreads employed in the empirical analyzes here conducted

		(AAA) – (A-)			(BBB+) – (BBB-)			(BB+) – (CCC)		
		2020	2019	2018	2020	2019	2018	2020	2019	2018
1 yr spread (%)	average	0.1042	0.1405	0.0932	0.1789	0.2841	0.1420	2.2272	1.3618	0.8167
	min	0.0292	0.0409	0.0199	0.0338	0.0489	0.0122	0.0858	0.0508	0.0505
	max	0.6314	0.5811	0.5504	1.3142	1.9967	1.3013	12.7028	10.9834	9.8224
	SD	0.1012	0.0956	0.0868	0.2041	0.3747	0.1754	3.6774	2.6002	2.3275
5 yr spread (%)	average	0.3301	0.4866	0.3188	0.6419	0.9448	0.5697	3.5204	3.1071	1.7693
	min	0.1364	0.1509	0.1326	0.1858	0.2837	0.2455	0.6927	0.8034	0.5895
	max	1.0003	1.0739	0.9008	1.9744	2.7815	1.7162	13.0551	7.6227	6.4411
	SD	0.1549	0.2005	0.1362	0.3509	0.5634	0.2653	3.3901	2.0977	1.5437
10 yr spread (%)	average	0.5897	0.7810	0.5828	1.0535	1.3542	0.9601	3.9485	3.7560	2.3981
	min	0.3066	0.3304	0.2879	0.4349	0.5766	0.5143	1.0073	0.9728	1.0096
	max	1.1963	1.5169	1.0982	2.4417	3.0960	3.0419	11.6705	8.4380	5.7303
	SD	0.2043	0.2591	0.1742	0.4245	0.5857	0.3757	2.9486	2.0516	1.4841
Curve slope (%)	average	0.0539	0.0712	0.0544	0.0972	0.1189	0.0909	0.1913	0.2660	0.1757
	min	0.0087	0.0251	0.0250	0.0025	0.0033	0.0037	− 0.4936	− 0.5850	− 0.4547
	max	0.1075	0.1314	0.0934	0.2315	0.2758	0.2975	0.5108	0.8054	0.4962
	SD	0.0196	0.0241	0.0160	0.0425	0.0486	0.0397	0.2415	0.2893	0.2005
Num. of companies		57			68			17		

The CDS spreads used for the calibrations are quoted on February 3, 2020, on February 1, 2019, and on February 1, 2018. Companies are organized in three groups according to their S&P rating. The first group is made of 57 companies characterized by medium and high investment grade, that is credit rating greater than or equal to $A-$. The second group is made of 68 companies characterized by low investment grade, that is credit rating in the range $BBB+$ and $BBB-$. The third group is made of 17 companies characterized by speculative (no investment) grade, that is credit rating lower than $BBB-$. The descriptive statistics are available for each one of these three groups. Curve slope is computed as: $\frac{10 \text {yrs spread} - 1 \text {yr spread}}{10-1}$

**Table 7:** Calibration of formulas $\Pi _{CIR}$ and $\Pi _{V}$ for a selected group of companies

Rating	Company	Model	$\frac{x_{0}}{x_{L}}$	$\alpha$	$\sigma _{x}$	*a*	*b*	MAPE %
AA	Allianz SE	$\Pi _{CIR}$	3.505	0.064	0.247	0.010	1.673	$9.16\%$
		$\Pi _{V}$	3.506	0.064	0.247	0.010	1.665	$9.18\%$
AA-	Shell PLC	$\Pi _{CIR}$	2.941	0.041	0.219	0.010	1.790	$11.20\%$
		$\Pi _{V}$	2.940	0.041	0.219	0.010	1.775	$11.22\%$
A+	BNP Paribas SA	$\Pi _{CIR}$	4.405	0.086	0.300	0.010	1.647	$10.74\%$
		$\Pi _{V}$	4.406	0.086	0.300	0.010	1.634	$10.75\%$
A	Banco Santander SA	$\Pi _{CIR}$	2.743	0.052	0.214	0.010	1.669	$6.68\%$
		$\Pi _{V}$	2.743	0.052	0.214	0.010	1.660	$6.70\%$
A-	Daimler AG	$\Pi _{CIR}$	2.600	0.023	0.210	0.010	1.456	$6.63\%$
		$\Pi _{V}$	2.600	0.023	0.210	0.010	1.443	$6.65\%$
BBB+	Deutsche Telekom AG	$\Pi _{CIR}$	3.860	0.074	0.292	0.010	1.690	$7.58\%$
		$\Pi _{V}$	3.860	0.074	0.292	0.010	1.683	$7.59\%$
BBB	Banco Sabadell SA	$\Pi _{CIR}$	2.929	0.042	0.262	0.010	1.350	$9.37\%$
		$\Pi _{V}$	2.927	0.042	0.262	0.010	1.345	$9.38\%$
BBB-	Edison SpA	$\Pi _{CIR}$	2.600	0.042	0.216	0.010	1.583	$4.60\%$
		$\Pi _{V}$	2.599	0.042	0.216	0.010	1.573	$4.60\%$
BB+	Leonardo SpA	$\Pi _{CIR}$	2.710	0.020	0.237	0.010	1.600	$7.55\%$
		$\Pi _{V}$	2.712	0.020	0.237	0.010	1.601	$7.54\%$
B	Selecta BV	$\Pi _{CIR}$	2.441	$-0.006$	0.265	0.010	0.629	$4.84\%$
		$\Pi _{V}$	2.444	$-0.006$	0.266	0.010	0.686	$4.85\%$

The calibration is performed by fitting the curves of CDS spreads. Maturities: 6 months and 1, 2, 3, 5, 7, 10, 20 and 30 years. The CDS spreads used are quoted on February 3, 2020. The CDSs employed have the following features. Restructuring clause: XR14. Seniority: Senior unsecured. The rating is by S&P. $\Pi _{CIR}$ indicates the credit risk model in Cathcart and El-Jahel (2003), that is theoretical values obtained using formula (22). $\Pi _{V}$ indicates the valuation framework here proposed, that is theoretical values obtained using formula (21). Thomson Reuters Eikon is our data provider. The calibrated values for all the other companies considered in the analysis are not reported for brevity, and are available upon request

**Table 8:** Calibration of the credit risk model here proposed (formula $\Pi _{_{V}}$) for the years 2018, 2019, and 2020, for a selected group of companies

Rating	Company	Model	$\frac{x_{0}}{x_{L}}$	$\alpha$	$\sigma _{x}$	*a*	*b*	MAPE %
AA	Allianz SE	2020	3.506	0.064	0.247	0.010	1.665	$9.18\%$
		2019	3.500	0.069	0.245	0.010	2.535	$3.99\%$
		2018	2.980	0.032	0.185	0.005	1.295	$5.46\%$
AA-	Shell PLC	2020	2.940	0.041	0.219	0.010	1.775	$11.22\%$
		2019	3.396	0.027	0.229	0.008	2.027	$2.79\%$
		2018	3.506	0.031	0.234	0.003	0.671	$4.05\%$
A+	BNP Paribas SA	2020	4.406	0.086	0.300	0.010	1.634	$10.75\%$
		2019	3.857	0.068	0.297	0.010	2.068	$7.41\%$
		2018	3.112	0.038	0.200	0.006	1.467	$1.80\%$
A	Banco Santander SA	2020	2.743	0.052	0.214	0.010	1.660	$6.70\%$
		2019	2.793	0.038	0.228	0.010	2.135	$6.58\%$
		2018	3.438	0.055	0.229	0.009	2.129	$1.62\%$
A-	Daimler AG	2020	2.600	0.023	0.210	0.010	1.443	$6.65\%$
		2019	2.500	0.031	0.216	0.010	2.049	$6.23\%$
		2018	3.083	0.041	0.217	0.009	2.429	$6.10\%$
BBB+	Deutsche Telekom AG	2020	3.860	0.074	0.292	0.010	1.683	$7.59\%$
		2019	4.108	0.072	0.297	0.010	2.423	$3.73\%$
		2018	4.518	0.067	0.285	0.008	2.004	$9.85\%$
BBB	Banco Sabadell SA	2020	2.927	0.042	0.262	0.010	1.345	$9.38\%$
		2019	3.241	0.039	0.288	0.010	0.662	$4.99\%$
		2018	3.216	0.052	0.254	0.010	2.039	$5.80\%$
BBB-	Edison SpA	2020	2.599	0.042	0.216	0.010	1.573	$4.60\%$
		2019	2.466	0.041	0.209	0.010	2.238	$3.13\%$
		2018	3.449	0.064	0.289	0.010	2.236	$5.13\%$
BB+	Leonardo SpA	2020	2.712	0.020	0.237	0.010	1.601	$7.54\%$
		2019	2.729	0.013	0.254	0.010	1.988	$6.65\%$
		2018	2.878	0.032	0.259	0.010	2.301	$4.73\%$
B	Selecta BV	2020	2.444	$-0.006$	0.266	0.010	0.383	$4.85\%$
		2019	2.595	$-0.026$	0.273	0.010	0.739	$5.51\%$
		2018	2.491	$-0.026$	0.265	0.010	0.410	$5.06\%$

The calibration is performed by fitting the curve of CDS spreads using formula (21). Maturities: 6 months and 1, 2, 3, 5, 7, 10, 20 and 30 years. The CDS spreads used are quoted on February 3, 2020, on February 1, 2019, and on February 1, 2018. The CDSs employed have the following features. Restructuring clause: XR14. Seniority: Senior unsecured. The rating is by S&P. Thomson Reuters Eikon is our data provider. The calibrated values for all the other companies considered in the analysis are not reported for brevity, and are available upon request

**Table 9:** List of the European companies to which the CDS spreads used for the calibrations refer to and their S&P’s credit rating

Company	S&P’s rating	Company	S&P’s rating	Company	S&P’s rating
Allianz SE	AA	Gecina SA	A-	Ahold Delhaize NV	BBB
Deutsche Bahn AG	AA	ING Groep NV	A-	Lafarge SA	BBB
Sanofi SA	AA	Kering SA	A-	LANXESS AG	BBB
apoBank eG	AA-	Klepierre SA	A-	Mediobanca SpA	BBB
DZ BANK AG	AA-	Koninklijke DSM NV	A-	Naturgy SA	BBB
Hannover RE	AA-	Schneider Electric SE	A-	NXP BV	BBB
Munich Re	AA-	Sodexo SA	A-	PostNL NV	BBB
Shell PLC	AA-	Thales SA	A-	Publicis Groupe SA	BBB
Scor SE	AA-	Vinci SA	A-	Repsol SA	BBB
Airbus SE	A+	Akzo Nobel NV	BBB+	Telefonica Europe BV	BBB
BMW AG	A+	AIB PLC	BBB+	Telefonica SA	BBB
BNP Fortis SA	A+	BNL SpA	BBB+	UniCredit SpA	BBB
BNP Paribas SA	A+	Bankinter SA	BBB+	Veolia SA	BBB
Rabobank NV	A+	Bertelsmann SE	BBB+	Vivendi SA	BBB
Credit Agricole CIB	A+	Capgemini SE	BBB+	Wendel SE	BBB
Credit Agricole SA	A+	Continental AG	BBB+	Abertis SA	BBB-
Credit Lyonnais SA	A+	Danone SA	BBB+	Accor SA	BBB-
ING Bank NV	A+	Deutsche Bank AG	BBB+	Auchan SA	BBB-
KBC Bank NV	A+	Deutsche Telekom AG	BBB+	Edison SpA	BBB-
LVMH SE	A+	Endesa SA	BBB+	EDP SA	BBB-
Siemens AG	A+	Enel SpA	BBB+	EDP Finance BV	BBB-
Total SA	A+	Heineken NV	BBB+	HeidelbergCement AG	BBB-
Unilever NV	A+	Iberdrola SA	BBB+	OTE SA	BBB-
AXA SA	A	Philips NV	BBB+	Peugeot SA	BBB-
Banco Santander SA	A	NN Group NV	BBB+	Renault SA	BBB-
BFCM SA	A	Orange SA	BBB+	Schaeffler AG	BBB-
BASF SE	A	Pernod Ricard SA	BBB+	Suedzucker AG	BBB-
Erste Bank AG	A	RBI AG	BBB+	UBI Banca SpA	BBB-
Henkel AG	A	Telekom Austria AG	BBB+	Valeo SA	BBB-
Helaba	A	TF1 SA	BBB+	Leonardo SpA	BB+
Linde AG	A	Terna SpA	BBB+	Smurfit Kappa PLC	BB+
Merck KGaA	A	HVB AG	BBB+	TIM SpA	BB+
Societe Generale SA	A	Volkswagen AG	BBB+	Millennium BCP SA	BB
Wfd Unibail-Rodamco SE	A	VWFS AG	BBB+	INEOS Group SA	BB
Aegon NV	A-	Wolters Kluwer NV	BBB+	Nielsen Co BV	BB
Air Liquide SA	A-	Banco Sabadell SA	BBB	Rexel SA	BB
AB InBev NV	A-	Bankia SA	BBB	Atlantia SpA	BB-
ASF SA	A-	Bayer AG	BBB	EUCAR SA	BB-
BBVA SA	A-	Carrefour SA	BBB	Thyssenkrupp AG	BB-
Bank of Ireland PLC	A-	Saint-Gobain SA	BBB	TUI AG	BB-
Bouygues SA	A-	Lufthansa AG	BBB	UPC Holding BV	BB-
Commerzbank AG	A-	DCL SA	BBB	CMA CGM SA	B+
Michelin SCA	A-	DVB Bank SE	BBB	Alpha Bank SA	B
Daimler AG	A-	E.ON SE	BBB	Groupe Casino SA	B
EDF SA	A-	Fresenius SE	BBB	Novafives SAS	B
EnBW AG	A-	Hochtief AG	BBB	Selecta BV	B
Engie SA	A-	Infineon Technologies AG	BBB		
Eni SpA	A-	Intesa Sanpaolo SpA	BBB		

## Appendix C

Table 6 reports the descriptive statistics for the CDS spreads considered. As expected, spreads tend to be higher for companies with worse credit rating, and on average were the highest in 2019. Considering the spread curve, spreads are typically higher for longer maturities indicating a positively sloped curve. The results of the calibrations for a selection of companies is available in Tables 7 and 8. Specifically, Table 7 shows the results of the calibrations (using quotations on February 3, 2020) for CDS pricing formulas (21) and (22), while Table 8 shows the calibrations for CDS pricing formula (21) in three different days, that is on February 3, 2020, on February 1, 2019, and on February 1, 2018. Finally, Table 9 reports the list of the 142 companies considered in the study.

## Footnotes

1 This allows us to capture the so-called *leverage effect*, that is value and volatility of equity are negatively correlated. However, Das and Sundaram (2007) conclude that the leverage effect has a limited impact on the term structure of default swap spreads and a geometric Brownian motion can be used to approximate firm’s equity value.

2 The CIR process was preferred to the Vasicek process till the 2007-2008 financial crisis. Afterwards, the observation of negative interest rates brought Vasicek model back into vogue. Indeed, differently from the CIR model, the Vasicek model allows to have negative interest rates with positive probability.

3 It can be considered a proxy of the firm’s equity value.

4 Only CDSs with maturities lower than or equal to 30 years are considered in the empirical analysis that follows and in general CDSs with maturities higher than 30 years are not traded. Concerning the order of approximation, the numerical test conducted, which is not reported here for the sake of space, indicates a computational error lower than $10^{-4}$. Since a CDS spread is measured in basis points, computing a CDS pricing formula with a level of precision of $10^{-4}$ is enough for the purpose of our application.

5 For the sake of comparison, the CDS pricing formula derived in Ballestra et al. (2020) for the valuation framework in Cathcart and El-Jahel (2003) is rewritten in (22) in terms of the price of a default-free bond, the current price of survival security and the forward price of a survival security, see appendix A.

6 This is consistent with the so-called *leverage effect*, according to which the volatility of a firm’s equity value is inversely related to the value of the equity itself, see, e.g., Das and Sundaram (2007). A property that is also inherent in the structural models, such as the ones in Merton (1974) and in Longstaff and Schwartz (1995).

7 The positive relationship between credit spread and risk-free rate observed for high rated (investment grade) companies is apparently inconsistent with the predictions of structural models, see, e.g., Merton (1974) and Longstaff and Schwartz (1995). However, this difference relay on the fact that structural models consider the *direct effect*, i.e. these models are based on the assumption that the growth rate of the value of the assets of a firm is proportional to the risk-free rate. On the contrary, the current modeling framework is based on the assumption that the credit quality signaling variable is not correlated to the risk-free interest rate. Therefore, only the indirect effect of the interest rate is considered, i.e. its role as a *discount factor*, which justifies a positive relationship between credit spreads and risk-free rates. Our results are consistent with the ones in Ballestra et al. (2020) and with the empirical evidence of a positive relationship between credit spreads and risk-free rates over the long-run documented in Morris et al. (1998).
